# How to Tackle Bacteriophages: The Review of Approaches with Mechanistic Insight

**DOI:** 10.3390/ijms24054447

**Published:** 2023-02-23

**Authors:** Monika Karczewska, Patryk Strzelecki, Agnieszka Szalewska-Pałasz, Dariusz Nowicki

**Affiliations:** 1 Department of Bacterial Molecular Genetics, Faculty of Biology, University of Gdansk, Wita Stwosza 59, 80-308 Gdansk, Poland; 2 Institut de Physique et Chimie des Matériaux de Strasbourg, Université de Strasbourg, CNRS, UMR7504, 23 rue du Loess, CEDEX 2, F-67034 Strasbourg, France

**Keywords:** bacteriophages, contamination, eradication, phage decontamination, T4, phi6, phiX174, MS2, *Lactococcus*, lactic acid bacteria, *Escherichia coli*

## Abstract

Bacteriophage-based applications have a renaissance today, increasingly marking their use in industry, medicine, food processing, biotechnology, and more. However, phages are considered resistant to various harsh environmental conditions; besides, they are characterized by high intra-group variability. Phage-related contaminations may therefore pose new challenges in the future due to the wider use of phages in industry and health care. Therefore, in this review, we summarize the current knowledge of bacteriophage disinfection methods, as well as highlight new technologies and approaches. We discuss the need for systematic solutions to improve bacteriophage control, taking into account their structural and environmental diversity.

## 1. Introduction

Bacteriophages (so-called phages) are considered the most diverse and abundant biological entities in the biosphere [1]. The estimated number of bacteriophage particles in nature is around 10^31^. Thereby, the relevant role of these viruses, which infect bacterial cells, cannot be neglected in several processes: (i) global ecology by controlling microbial population sizes; (ii) microbial evolution by promoting diversification and genetic transfer; (iii) scientific research, serving as models in molecular biology and providing experimental tools for analysis and manipulation of host cells at the molecular level; (iv) health system, as tools to control of microbial infections [2,3,4,5]. On the other hand, they are of great concern to the industry due to their negative impact on biofermentation processes, e.g., protein synthesis or the dairy industry [2,4,6,7]. Their role in microbial pathogenesis, as carriers of virulence genes transmission, is an ongoing challenge for the health care system.

Phages can be classified based on their shape, genetic material, and mode of infection [8]. They can also be grouped into families based on shared genetic and structural features (Table 1). They are composed of a protein capsid that surrounds their genetic material, which can be either DNA or RNA. The capsid is often spherical or elongated in shape and can vary in size depending on the species of bacteriophage. Some bacteriophages also have additional structures, such as tail or tail fibers, that help them attach to and enter bacterial cells. Like all viruses, bacteriophages are very species-specific with regard to their hosts and usually only infect a single bacterial species or even specific strains within a species. Furthermore, when a phage attacks its prey, it can carry out either only a lytic or both lytic and lysogenic life cycle, whereby lytic phages kill host cells and lysogenic phages incorporate their genetic material into the host-cell’s genome [9,10]. The genetic material of bacteriophages is highly variable and can be replicated within the host cell, allowing the virus to reproduce and infect other bacteria. Once phage infects the host cell, it hijacks the bacteria’s metabolic pathways in order to propagate its particles.

Bacteriophages have been used in medicine and biotechnology, including the development of bio-sensors, vaccines, antibiotics, and as a potential alternative to drugs in the treatment of bacterial infections and biofilms [11,12,13,14,15]. However, the knowledge of phage biology, especially for the model viruses, is well established and assumes their relevant contribution to antimicrobial resistance spreading among microbes [16,17,18]. Transduction by bacteriophages is one of many horizontal gene transfer mechanisms that promote genetic variation. While transmission of chromosomal DNA as a result of generalized transduction remains a rare phenomenon (approximately once in every 10^7^–10^9^ phage infections), the sheer abundance of existing phages and bacteria renders this process extremely frequent [19]. Even more importantly, this process relates to the spread of virulence genes and antimicrobial resistance [20,21,22,23]. According to the current knowledge, phage-related virulence of pathogenic bacteria involves classical type I membrane-acting superantigens, type II pore-forming lysins, and type III exotoxins, such as diphtheria and botulinum toxins as well as Shiga toxin. The uncontrolled and inappropriate use of bacteriophages capable of gene transduction can pose a threat to human life and health, and international initiatives have therefore established guidelines for the use of phages in therapy [24,25]. To date, disinfection methods and standards focused on the eradication of bacterial pathogens, but not bacteriophages whose distribution in medical and industrial environments is not sufficiently controlled.

This review discusses various methods that so far have been developed to eradicate phages, from physical decontamination to controlling viral development within the host cell, with respect to their molecular basis. Some of these methods are universally employed in sterilization and sanitation processes to eradicate a broad spectrum of microorganisms. Typically, these approaches are tested on the model bacteria and viruses, which cannot be adequate for all pathogens. Moreover, we note that while phages have been traditionally seen as natural means of controlling bacterial populations, their ability to mutate and adapt to new environments has raised concerns about their potential to spread in environments where their presence is considered undesirable. Therefore, we state that a more systematic approach is needed to develop effective solutions to control phage spreading. 

## 2. Physical Methods Used to Inactivate Bacteriophages

The risk of bacteriophage infection can be reduced by several techniques, including sterilization by physical agents. We examined existing databases for physical factors affecting the stability of bacteriophages (Table 2), and in this chapter, we present the main methods used to inactivate them. We focus on the use of conventional techniques such as disinfection by heat, pressure, humidity, and UV light. The last method has become a rapidly developing chemical-free technology in recent decades. We pay particular attention to the use of filtration and to newly developed technologies with two important ones: non-thermal plasma processes and laser technologies.

### 2.1. Thermal Disinfection

Temperature regulation is a well-known method that has been used for decades or even centuries as the main method of environmental microorganism inactivation; also, it is widely used in the food industry [26]. Most bacteriophage inactivation research is focused on the application of thermal disinfection [27,28,29,30,31,32,33,34,35,36,37,38,39,40,41,42,43,44]. Additionally, when using microwave radiation, it is the thermal effect that is associated with the inactivation of bacteriophages, compared to the application of radiation under non-thermal conditions [45]. Such conclusions were reached by Bryant et al. in an attempt to explain the mechanism of inactivation by microwave radiation of bacteriophage T4 that occurs within 20 s when compared to control samples treated on ice [45]. The mechanism of inactivation is most likely related to damage of the capsid, but before reaching the melting point, DNA is released [39,40]. The most studied application of this disinfection method is the control of bacteriophages infecting lactic acid bacteria (LAB) [46]. Among these bacteriophages, there are some that can survive pasteurization due to their high heat resistance, e.g., P680, P1532, and P008 [31,33,47]. Another interesting finding from these studies is the importance of the culture medium and its composition for bacteriophage inactivation efficiency. In the presence of fat, phage survival increases which is related to its protective effect by keeping the particles moist [27,28,29]. Such conclusions were reached, among others, by Muller-Merbach et al., who inactivated the model phage P008 in selective M17 broth and milk. In the case of M17 broth, the higher the temperature used, the faster the inactivation progressed. Exposure to 55 °C led to a 1-log reduction within 3 h. Under short-term pasteurization conditions (i.e., 30 s at 75 °C), about 1 log of the phage population was inactivated, with a 7-log decrease after 6 min at this temperature. In comparison, inactivation in milk proceeded more slowly. At 55 °C, the phage titer hardly dropped even after 24 h, and short pasteurization conditions reduced the P008 titer by less than 1 log [27,28,29].

### 2.2. UV Radiation 

UV radiation has been a validated technology for disinfecting surfaces as well as in air and water. It can eradicate a wide range of microorganisms. UV radiation is becoming an increasingly affordable method that yields reproducible significant reductions of infection [48,49]. Factors that may be involved in phage susceptibility to UV wavelengths are the type of nucleic acids (DNA or RNA), genome structure (single- or double-stranded), guanine and cytosine content, lipid envelope, the size of the viral particle, as well as other features of molecular structure. Therefore, in general, bacteriophages containing single-stranded RNA or DNA are more sensitive to UV radiation than phages containing double-stranded RNA or DNA. Tseng et al. determined in their study that the UV dose causing 99% inactivation was twice as high for phages containing ssRNA/DNA (MS2 and ΦX-174, respectively) than for dsRNA/DNA (Φ6 and T7, respectively) [50]. For all four virus types, the survival fraction decreased exponentially with increasing dose, by either increasing the UV intensity or exposure time. Toxic UV photoproducts are usually thymine dimers, so RNA viruses are more resistant to UV damage than DNA viruses [51], with the UV dose causing 99.9% (4 log) reduction in bacteriophages for MS2 (RNA) versus PRD1 (DNA) was 65.2 and 31.6 mW/cm^2^, respectively. Similar results were observed [51,52,53,54,55,56] when MS2 or Qβ phage (RNA) was compared with ΦX-174 (DNA), obtaining results with higher UV sensitivity for DNA bacteriophage. Therefore, each bacteriophage may have different susceptibility to UV dose, and this affects the effectiveness of the UV disinfection [57,58,59]. Ultraviolet waves spectra are not exclusive for inactivation of bacteriophages. Several reports demonstrating phage sensitivity to visible light (VL) at 405 and 455 nm have been published [60,61,62]. Inactivation of microorganisms under visible light can be associated with photodynamic inactivation (PDI) where a photosensitizer is excited by specific wavelengths of visible light in the presence of oxygen that leads to the production of reactive oxygen species (ROS), ultimately resulting in structural damage. Tomb and colleagues studied the effect of violet-blue light on the reduction of phage ΦC31 (genetic material on form of dsDNA) [61]. For the 10^3^ PFU/mL, they achieved a 2.7 log reduction after exposure to 0.3 kJ/cm^2^, while ΦC31 titer of 10^5^ and 10^7^ PFU/mL were successfully decreased by ~5- and 7 log after exposure to doses of 0.5 and 1.4 kJ/cm^2^, respectively, by 405 nm light. It should be noted here that the inactivation was effective if the phage was suspended in liquids or substrates containing appropriate light-sensitive components (photosensitive porphyrin molecules), while no reduction in phage titer was observed when suspended in PBS. However, the study by Vatter et al. demonstrated inactivation of the enveloped virus Φ6 at 7.2 kJ/cm^2^ [60]. The phage titer was reduced by more than three folds within 40 h without the addition of photosensitizers [60]. However, Φ6 phage differs in genetic material structure (dsRNA) and the presence of an envelope, which is in line with previous reports that the structure of a bacteriophage affects the conditions of the observed inactivation efficiency.

Phage-inactivating agents can also be used in combination with other technologies to increase disinfection efficiency, so the use of UV or visible light with ultrasound (US) shows synergistic effects. This has been proven by the study in which the simultaneous application of US and VL was more effective than US alone for MS2 inactivation [63]. Moreover, along with UV light, synergy has been shown in combination with US (bacteriophage from Klip river) [64], ozone (MS2 bacteriophage) [65], or silver ions (MS2 bacteriophage) [66].

### 2.3. Pressure and Humidity

The effect of pressure on bacteriophages appears to be effective at values greater than 300 MPa [67,68]; this has been particularly studied for lactic acid bacterial phages, which were resistant to pressure ≤100 MPa [69,70,71,72]. Electron microscope images showed shrunken phage heads containing or lacking DNA after applying pressure on T4 phage [73]. 

The least effective appears to be the impact of humidity, since many additional factors affect its efficiency, such as the structure of the bacteriophage. The survival rate of the non-sheath phage MS2 turns out to be better than that of the enveloped phage Φ6 [74]. The pH, presence of proteins and environmental factors also have an impact of phage sensitivity. Bacteriophages survive in the range of low and high values of relative humidity, which in addition is often correlated with temperature, and only the intermediate value of humidity is effective in virus eradication, which is also dependent on the phage type. While salt, pH and surfactant reduced survival under wide range of humidity conditions, proteins provided some protection against phage particles degradation [74,75,76,77,78,79,80,81]. Thus, the effect of chemical composition has a significant impact on relative humidity effectiveness, highlighting the importance of simultaneous investigation of different factors in bacteriophage survival.

### 2.4. Filtration

Filtration technology is not a new invention; however, due to a rapid development through modifications of membrane elements, it has been continuously improved in terms of performance over past 50 years. New materials with improved chemical and thermo-mechanical properties and better permeability and selectivity are increasingly applied. The development of membranes significantly increases the range of applications of filtration, hence in the literature one can find many studies on the use of the technique in industry, which includes purification of water and dairy products as well as wastewater and air. It is also being used in the production processes, the environment, and public health applications [82,83]. The rapid development of nanotechnology has sparked great interest in nanomaterials, which are excellent adsorbents, catalysts and sensors due to their large specific surface area and high reactivity. Several natural nanomaterials have been shown to have strong antimicrobial properties. These include, for example, carbon nanotubes (CNTs), which can enhance membrane filtration [84,85]. CNTs are graphene sheets, either single-walled (SWNT—single tube) or multi-walled (MWNT—several packed tubes) [86]. Research by Brady-Estevez et al. has shown that bacteriophages are removed by the CNT filter matrix through a deep filtration mechanism, that is, captured by bundles of nanotubes inside the SWNT layer [87]. The filter was developed using a microporous poly(vinylidene fluoride) (PVDF)-based membrane coated with a thin layer of SWNTs. A model virus particle, bacteriophage MS2, with a diameter of 27 nm, was employed and the results indicated complete removal of bacteriophage particles. This thickness of the SWNT layer removes 10^7^ virus particles per mL (5–7 log) [87]. However, the removal of MS2 bacteriophages by the MWNT filter was 1.5 to 3 log higher than that observed in SWNTs [88]. Brady-Estevez et al. also determined the efficiency of the SWNT-MWNT hybrid layer on different bacteriophages, i.e., MS2, PRD1 and T4, which have different structures, ribonucleic acids, diameters, and isoelectric points [89]. The hybrid filter was expected to be more similar to the performance of the MWNT filter, since the nanotubes were made of 83% MWNT and only 17% SWNT, and SWNT alone had a much lower efficiency. However, the SWNT–MWNT dual filter performed better than the 100% MWNT filter, and is effective against a wide range of bacteriophages [89]. Nevertheless, the complex chemical compositions of solutions and the presence of impurities can affect filter performance. Phage removal increased at higher ionic strengths (NaCl) due to suppression of repulsive electrostatic interactions between viruses and nanotubes. The addition of divalent salts, on the other hand, had opposite effects. While CaCl_2_ increased the removal, probably due to the complexation of calcium ions with the phage surface, the addition of MgCl_2_ decreased the phage eradication [90]. This effect was also observed in other cases, and it was determined that SJC3 phage filtration was strongly dependent on the concentration and valence of the dominant cation in the pore fluid. While using a filtration system consisting of quartz sand-filled columns, column retention increased from 0% to 99.99% when the electrolyte composition was changed from NaCl to CaCl_2_ [91].

### 2.5. Femtosecond Laser

Another modern technique is femtosecond laser irradiation. These are ultra-short laser pulses that show great potential for disinfection. Work by Tsen et al. has shown that femtosecond infrared and visible lasers can inactivate phages, and they attribute this to a mechanism called pulsed stimulated Raman scattering (ISRS) [92,93,94,95,96,97,98,99]. It appears that during ISRS, vibrational excitation of the capsid and disruption of the protein coat occur. The sample’s exposure time to laser radiation in the study by Tsen et al. was about 1 h or longer and resulted in a 5-log reduction of M13 phage titer [95]. Gel electrophoresis results indicated that laser irradiation does not change the structure of single-stranded DNA but leads to the breaking of hydrogen/hydrophobic bonds or the separation of weak protein linkages in the envelope [95,98]. More recently, Berchtikou et al. used millijoule laser pulses (40 fs) with different exposure times (1–15 min) and different wavelengths (800, 400 nm separately of combined), pulse energy ~20 mJ, and repetition rate of 10 Hz [96]. According to data presented, the 4-log reduction of phage titer took 31 min with 800 nm wavelength of laser used. Further evaluation showed that longer exposure times and shorter excitation wavelengths result in greater reduction of viral counts. The maximum observed inactivation about 6 log was obtained using a femtosecond laser with a wavelength of 400 nm, energy of 20 mJ, and pulse width of 40 fs, after 15 min of exposure. The authors deduced that virus inactivation increases with increasing irradiation energy density and shortening wavelength [100]. 

### 2.6. Non-Thermal Plasma

A promising approach to sterilization and disinfection is the use of atmospheric pressure non-thermal plasma (APNTP). APNTP has potential advantages over standard chemical disinfectants and sanitizers. First of all, it uses non-toxic gases and is known for the absence of toxic products during its process. The effectiveness of disinfection is related to the generation of a large number of different active agents, including chemically reactive forms (oxygen and nitrogen), UV or electromagnetic fields [101]. There are several reports on the effectiveness of APNTPs in inactivating bacteriophages. Venezia et al. obtained a reduction in the PFU/mL of λ C-17 and lytic bacteriophage (Rambo; Microphage) by at least 4–6 logs after 10 min of exposure [102]. On the other hand, Yasuda et al. observed inactivation of λ phage by 6 logs after 20 s using stable plasma generated by dielectric barrier discharge (DBD) [103]. Both of these studies detected nucleic acid damage, as well as changes in coat proteins. During the investigation what factors could improve the efficiency of inactivation by plasma, it was found that the percentage concentration of oxygen in the carrier gas was positively correlated with the rate of phage inactivation (MS2). Namely, oxygen concentration (0.75%) and 3 min of exposure to a plasma source operating in a helium/oxygen gas mixture (99.25%:0.75%) resulted in 99.9% reduction of MS2, additionally, increasing the time to 9 min resulted in >7 log inactivation. Moreover, interesting results of pre-activation of water with plasma were also presented. Water was pre-treated with plasma (for 120 s for T4 or 80 s for Φ174 and MS2) and then mixed with suspensions of tested bacteriophages. After incubation for 4 and 8 h with such prepared water, the titer of bacteriophage T4 was reduced by about 7.2 and 8.8 orders of magnitude, respectively, indicating that the process was time-dependent. The titers of active bacteriophages Φ174 and MS2 decreased close to the detection limit. Moreover, the action of plasma alone for 100 s completely abolished the infectivity of bacteriophage T4 suspension, and a similar effect for the other two phages was obtained after 60 s [104].

**Table 2:** The physical methods to eradicate bacteriophages.

Factor	Phage	Conditions	Remarks/Mechanism	References
Temperature	P008, pll98, MS2, P680, P1532, PRD1, ΦX174, somatic coliphages, *Bacteroides fragilis* phage, *Lactobacillus helveticus*, *Lactococcus lactis* bacteriophages, OMKO1, HK97, λ, PP7, thermophilic *Bacillus* phages	Medium temperature range from 55 °C to 100 °C	Structural damage, protein denaturation The medium plays an important role in terms of the thermal resistance of phages P680 requires a higher temperature (from 100 °C for 20 min to 140 °C for 2 s)	[27,28,29,30,31,32,33,34,35,36,37,38,39,40,41,42,43,44]
	*E. coli* phage	Inactivation in the wet and dry state	In the dried state, rate of inactivation varies exponentially	[105]
	MS2	Low temperature 4–15 °C	Reduction 15 °C after 30 days Virus inactivation of 2 log at 15 °C after 30 days and reduction of 3.5 log at 25 °C after 28 days	[106,107]
Pressure	832-B1, QP4, QF12, 13.2, B1, MLC-A, MLC-A8, Φi*Lp*84, Φi*Lp*1308	<100 MPa	High pressure resistance	[69,70]
	P001, P008	0.1–600 MPa 25–80 °C	Structural damage caused by pressure and heat combination. However, over a specific range of pressure and heat, they act antagonistically	[71,72]
	ΦX174, λ,T4, MS2	>300 MPa	Structural damage caused by pressure: (1) phage with shrunken envelopes and DNA-containing heads; (2) phage with shrunken envelopes and heads lacking DNA. The ratio of the two types is strongly dependent on temperature used	[67,68,73,108]
Irradiation	MS2, S-13, C-36 and Staph-K, ΦX-174, B40-8	y-rays, X-rays and a-rays	Dose effect dependent on exposure time	[109,110,111]
	Φ6	IR 0.5 m for 3 h at different humidity levels	Over 90% inactivation at humidity levels above 50%	[112]
Microwaves	T4, T7, λ, MS2, *E. coli* bacteriophage isolated from sewage	Different times from 10 s to 2 min	Thermal inactivation	[45,113,114,115]
Filtration	MS2	Modified Al_2_O_3_ granular ceramic filter materials Al_2_O_3_ or Cu/Ag	Highly porous granular structures play a key role in the removal	[116,117]
	SJC3	Columns of quartz sand	Filtration strongly dependent on the concentration and valence of the dominant cation in the pore fluid (CaCl_2_ increased virus removal)	[91]
	MS2, PRD1,T4	Carbon nanotubes (CNT)	Both filtration and inactivation of viral aerosols, CaCl_2_ increased virus removal, likely due to complexation of calcium ions to viral surface	[87,88,89,118]
	λ, T4, MS2	Iodinated resin filters	Structural damage to the capsid protein through filter enrichment with iodine	[119,120,121]
	f2, MS2, T4, T7	Filtration and UV		[122,123,124,125]
UV	λ, MS2, PRD1, R17, PP7, fd, M13, T4, T7, SP8, ΦX174, B40-8, GA, Qβ, Staphylococcus-phage A994, Φ6, P680, P008, T1, P22, T2, R17	From 9 mJ/cm^2^ to 50 mJ/cm^2^ depending on the phage	Time- and phage-dependent dose. MS2 phage had the greatest resistance	[53,54,55,56,57,110,126,127,128,129,130,131,132,133,134,135,136,137,138,139,140,141,142,143]
	MS2, different coliphages from the treated municipal wastewater	0.05–0.25 mg/L Cl and 14–22 mWs/cm^2^ UV	More effective than chlorine alone	[144,145,146]
Ozone	MS2	0.03 mg min/L and a small O_3_-Ct value	ROS-mediated oxidative damage. The synergistic effect after the sequential ozone-UV and UV-ozone exposures	[65,147,148]
Electric field	M13, M18, λ	Pulsed electric field (PEF), 5 or 7 kV	Survival ratios after 12 min PEF treatment were 10^−4^–10^−5^ inactivation regardless of the form of the phage particle	[149]
Ultrasound (US)	Phage of the *Bacillus megaterium*, bacteriophages in Klip River water, ΦX174, MS2	29.10, 582, 862, 1142 kHz	The synergistic effect US and UV	[63,64,150,151]
Plasma	ΦX174	One atmosphere uniform glow discharge plasma (OAUGDP)	Titer reduction >10^6^ after 15 min	[152,153]
	ΦX174, MS2, λ	Non-thermal atmospheric pressure plasma	Membrane destruction, inactivation of proteins, and DNA damage	[102,103,154,155,156,157,158]
	MS2	Nonthermal plasma jet operated at varying helium/oxygen	Inactivation is a function of oxygen concentration in the carrier gas mixture	[101,156]
	ΦX174, MS2, T4	Surface plasma in argon mixed with 1% air and plasma-activated water	ROS-mediated oxidative damage	[104]
Energetic femtosecond lasers	MS2, M13	400–800 nm lasers	Coats’ proteins disruption through laser-induced excitation of large-amplitude acoustic vibrations	[92,93,94,95,96,97,98,99,100,159,160]
Visible Light	ΦC31, Φ6	405, 455 nm	ROS-mediated oxidative damage	[60,61,62]
Humidity	MS2, Φ6, T3	Range from low to high RH	Structure damage	[74,75,76,78,79,80,81]

## 3. Bacteriophages Eradication Approaches Using Metals, Ions, and Other Inorganic Materials

Inorganic compounds have been known for their antiviral properties for centuries. Gases such as ozone [161] or carbon dioxide [162,163] and metals were studied for their ability to combat bacteriophages. Most common metals used in these processes are silver [164,165,166], copper [165,166,167], and iron [168,169,170,171,172]. Many of them and their oxides and salts have been extensively studied for their ability to inactivate series of different bacteriophages such as MS2, Φ6, Φ8, PP7, ΦX174, PM2, T4, T7, and Qβ. Those and other prominent agents used to inactivate bacteriophages are summarized in Table 3. With new emerging technologies and manufacturing techniques there are possibilities to create various combinations and modifications of metals that can provide new effective ways to combat viruses. One of the most promising fields is nanotechnology that allows to create nanoscale particles of metals that with unique properties differ from the input material that has been used to synthesize them. The most recent and innovative methods are described in the next sections. 

### 3.1. Nanoparticles 

Nanotechnology is an emerging field that gained significant attention in recent years. A series of nanoparticles (NPs) have been already developed with a variety of potential applications [173]. The most common group of nanoparticles are based on metal such as silver, copper and gold. The properties (size, shape, and coating) of NPs strongly determine their potential application. Currently, nanotechnology is being used in a range of areas such as the manufacturing of materials, electronics, energy harvesting, the mechanical industry as well as drugs and medications [174,175]. As their precursor metal NPs have the ability to inactivate pathogens so a series of works provided data on their ability to inactivate bacteriophage lysates. 

#### 3.1.1. Silver Nanoparticles

AgNPs are of the most promising class because of several properties such as electric conductivity, antimicrobial activity, high surface to volume ratio, swelling, and contraction flexibility [176]. Their ability to combat viruses is highly dependent on their size with smaller NP being the most effective [177]. One of the works by Gilcrease et al. from 2020 demonstrated that silver nanoparticles negatively affected phage lytic growth cycle [178]. In the experiments involving a series of bacteriophages RG2014, KL, Det7, P22, SP6, and 9NA, uncoated bare silver nanoparticles reduced infection yields of phage RG2014 by 89% and phage KL by 92.4% after 70 min of infection. Polyvinylpyrrolidone-coated (PVP) silver nanoparticles reduced the post infection PFU/mL of RG2014 by 74%. Interestingly, PVP-coated AgNPs increased the yield of phage KL by 92%. However, phages P22, 9NA, SP6, and SF6 were less sensitive to NPs action. It was then suggested that the exposed regions of the viral coat proteins of RG2014, KL, and Det7 may share nanoparticle binding features (strong enough to overcome the weaker repelling forces between the negatively charged surfaces of phage and nanoparticles) that other phages do not possess. Further studies showed that the difference between affected and unaffected phages lay in their structure, namely the presence of overhanging positively charged capsids’ protein C-terminus., which facilitated the binding of nanoparticles [178]. Moreover, according to presented data, AgNPs and their ions also significantly affected phages at concentrations and incubation times in culture low enough to not affect their host growth. This definitely increases the chances of exploiting NPs in, e.g., biofermentation processes.

The effectiveness of smaller nanoparticles has been confirmed in another work by Gokulan et al. from 2018 focused on MS2, PP7, and ΦX174 bacteriophages [179]. Effectiveness was dependent on the size and dose of NPs as well as on the temperature. All bacteriophages were more susceptible to the AgNP-mediated killing at 37 °C as compared to 4 °C. Exposure of MS2 phages to high dosage of AgNP at 37 °C resulted in the absence of PFU after 14 days, whereas at 4 °C, there was no difference in the PFU formation by MS2 during the treatment of any dose of AgNP; however, there was a clear difference between the control and AgNP-treated MS2 phages after day 2 (reduction of around 2–3 logs after 28 days). The PP7 phages appear to be more susceptible to AgNP at high and medium doses–these phages were completely inactivated at 37 °C after 14 days. At 4 °C, only 1–2-log decrease in the PFU/mL was observed [179]. All these data show that inactivation differs not only between bacteriophages but also depends on the time and temperature.

There are several reports that show potential of combining AgNPs with different materials to give them new unique properties. For example, glycoprotein, curcumin, and stabilizers can be added to AgNPs to improve their antimicrobial potential [180,181,182]. In the work from 2018 Park et al. designed a silica hybrid composite decorated with AgNPs [183]. The antiviral effect has been studied with the use of MS2 bacteriophage. After 24 h of exposure, phage titer was reduced more than a 3 log. Released Ag^+^ ions, originating in nanoparticles, can contribute to strong antiviral capabilities. AgNP could be easily recovered in water conditions via sedimentation or centrifugation, and in addition, these particles can be reused, which means that AgNP-SiO_2_ particles could be more effective and environmentally friendly tool to control waterborne viruses [183].

The effectiveness of NP-Ag-CuO was tested by Shimabuku et al. in the works published in 2017 and 2018 [184,185]. The antiviral activity has been checked with the use of the granular activated carbon (GAC) modified with silver and/or copper oxide nanoparticles. The porous media containing silver and copper oxide nanoparticles showed inactivation reaching reductions higher than 3 logs [185]. GAC filter itself has only a potential for a reduction of 0.32 log [186]. The presence of copper oxide nanoparticles did not improve the efficiency of virus particles inactivation. The use of 1% AgNP increased inactivation by 0.64 log. However, values higher than 3.02 log in PFU/mL reduction was showed for the combination of both silver and copper oxide mixtures. The most efficient combination (1% Ag and 1% Cu) reached a reduction value of 5.56 log. This effect is most likely caused by the released ions, ROS, or both [184,185]. 

#### 3.1.2. Gold Nanoparticles

A series of works focus on gold-based nanoparticles. One of them by Richter et al. from 2021 presents nanoparticles that deactivate bacteriophages and at the same time are safe for host bacteria [187]. It has been shown that AuNPs coated with a mixture of negatively charged 11-mercapto 1-undecanesulfonic acid (MUS) and hydrophobic 1-octanethiol (OT) ligands are effective in deactivating various types of *Escherichia coli* selective phages: T1, T4, and T7. The titer of phages can be lowered even to 2 logs in 6 h and 5 logs in 24 h. The most effective combination MUS:OT (85:15) required just a step of 1 h preincubation at 50 °C to fully deactivate T1 phages. MUS:OT nanoparticles were not effective against MS2 bacteriophages that lack the complex head-tail structure. The mechanisms of deactivation were based on initial electrostatic attraction followed by hydrophobic interactions causing local irreversible distortions in the phage heads [187].

#### 3.1.3. Nanoscale Zero-Valent Iron, iron, and Nickel Nanoparticles

Nanoscale zero-valent iron (nZVI) due to its size, surface effect and quantum size effect has a various applications including inactivation effect on bacteriophages [162,188,189,190,191]. In the study from 2018 by Cheng et al., the Fe/Ni nanoparticles (Fe/Ni NPs) and (nZVI) were assessed for their antiviral ability on f2 bacteriophages [190]. Fe/Ni NPs had higher deactivation efficiency and after 30 min of their action, bacteriophage was removed. It took 1 h for nZVI to reach the same point, while NiNPs showed no effect. Further studies showed that Ni^0^ in the Fe/Ni NPs facilitated the removal of phage f2 by induced production of ROS as a catalyst. To better understand the basis of this interaction, the influence of pH and oxygen was assessed. Efficiency was higher under aerobic conditions than that in the anaerobic system which can be connected to the fact that ROS generated from the oxidation of Fe^0^ and the catalysis of Ni^0^ are responsible for the inactivation mechanism. The changes in pH did not have an influence on effectiveness. As for changes in the temperature, it improves the reaction rate at the initial stage but decreases the removal efficiency due to the accelerated corrosion of iron. The inactivation mechanism of bacteriophage f2 by Fe/Ni NPs was related to the ROS generated from the oxidation of Fe^0^ and the catalysis of Ni^0^ [188,189,190]. Another work by Kim et al. from 2011 proved the effectiveness of nZVI on MS2 coliphage [172]. The inactivation of MS2 was much greater under air-saturated conditions (5.3 log) than under deaerated conditions (2.6 log). This is consistent with damage by reactive species formed via oxidation of nZVI. Unlike f2 phage, the inactivation of MS2 increased as pH decreased. The addition of 1,10-phenanthroline completely blocks oxidant formation. The reduction still occurred after the addition of this compound which proves that the mechanism of action must be also connected to the direct interaction and physical disruption caused by the nanoparticles [172].

Most recent study by Cheng et al. compared the influence of nZVI on MS2 and ΦX174 containing RNA and DNA, respectively [191]. It has been found that an initial concentration of 10^6^ PFU/mL of MS2 could be completely inactivated within 240 min, but the complete inactivation of ΦX174 could not be achieved by extending the reaction time, increasing the concentration, or changing the dosing means. Three-dimensional fluorescence spectrum and sodium dodecyl sulfate polyacrylamide gel electrophoresis (SDS-PAGE) have been used to examine the mechanism of nZVI action. The nucleic acid analysis demonstrated that the genome of MS2, but not ΦX174, was destroyed. It indicated that bacteriophage inactivation was mainly attributed to the damage of their genetic material [191].

Work from Raza et al. showed that the activity of zero-valent iron varies strongly against different phages and that different forms of ZVI, namely pristine (reduced) ZVI, PO ZVI (ZVI nanoparticles that were exposed to air and oxidized while incubated with phages), and O ZVI (oxidized ZVI, which was completely oxidized before the addition of phages) are also impacting bacteriophages in different ways [192]. M13 is very vulnerable to all studied forms of ZVI, whereas T7 appears almost completely resistant. T4 and T7 belong to the same class (*Caudoviricetes*), yet significant inactivation of T4 is observed. Pristine ZVI is active against M13 and T4, but not T7 and MS2. In the case of T4, PO ZVI shows much lower activity compared to ZVI and O ZVI. All of these findings show that when using nanoscale zero-valent iron to inactivate bacteriophages a series of factors need to be considered which limits the spectrum of these methods. 

### 3.2. Surface Coated Metal Inactivation

With the development of new techniques, materials processing has been significantly upgraded in recent years [193]. The use of, for instance, selective laser melting (SLM), aimed at using a high-power density laser to melt and fuse metallic powders, or plasma sintering (SPS), a synthesis technique that uses low voltage and direct current, has had a significant impact on producing a new range of materials and bringing them into wider use [194]. Materials with improved and more effective antiviral properties are intensively studied [195,196]. Rahmani and coworkers presented a method of phage inactivation based on the use of SLM and SPS to fabricate new materials, so-called metal matrix composites (MMC) [196]. Silver-doped titanium dioxide (TiO_2_ + 2.5–10% Ag), copper-doped titanium dioxide (TiO_2_ + 2.5–10% Cu), Cu_2_NiSiCr, Cu_15_Ni_8_Sn as well as pure copper spark plasma sintered discs were tested for their virucidal abilities on Φ6 bacteriophage. Phage inactivation on MMC surfaces corresponded to 99.99% and above was observed, and its effectiveness was related to the composition of the material used. Initial virus titer 10^10^ PFU/mL on the TiO_2_ + 10% Ag ceramic and CuNi_2_SiCr metal discs decreased by 4 logs after 15 min. Another work from the same team tested two different materials created with the use to the same technique to evaluate the effectiveness of 45% TiO_2_ + 5% Ag + 45% ZrO_2_ + 5% Cu and Co_28_Cr_6_Mo [195]. The two disks adsorbed all of the added virus suspensions during the 15-minute incubation. The surface infiltration time by the virus suspension was particularly short (3–5 s) on used Co_28_Cr_6_Mo metal disks. A total number of viruses attached to the disk, was still significantly higher than that in control steel disks, proving virucidal properties of studied material. When 10^8^ PFU were added to the 45% TiO_2_ + 5% Ag + 45% ZrO_2_ + 5% Cu disk surface, most viruses infiltrated the disk. The authors suggest that 99.99% of viruses, placed on the surface, were either irreversibly attached or inactivated, therefore possessing no threat to potential host cells. 

TiO_2_ photocatalyst has been proven to be effective in water disinfection [197]. In particular, this technique leads to generation of reactive oxygen species as a virucidal factor. Pure TiO_2_ can only be activated by light in the near UV range. To overcome this problem, researchers found that metal doping (e.g., V, Cr, Cu, Co, Ag, and Au.) is an effective method to extend the spectral response of TiO_2_ to the visible region, as well as decrease the electron-hole recombination rate [197,198,199,200,201]. Ditta et al. studied the photocatalytic activity of TiO_2-_, CuO and hybrid CuO/ TiO_2_ prepared by atmospheric Chemical Vapor Deposition (Ap-CVD) coated surfaces and TiO_2_ prepared by a sol–gel process against T4 bacteriophage [201]. Employed the sol–gel coated glass deactivation of virus particles by 6 logs was observed after 2–4 h. Moreover, they showed the improved results using CVD CuO coated samples. Efficiency of phage particle reduction by over 6 logs was obtained in shorter time (80 min). Furthermore, combination of TiO_2_ and CuO provided higher inactivation of >9 log after 80 min. The combination of photocatalysis and toxicity of copper acted synergistically to inactivate T4 bacteriophage [201]. In their work, Zheng et al. investigated the activity of prepared Cu-TiO_2_ nanofibers under visible light against bacteriophage f2 [202]. All viruses were inactivated within 240 min when the initial concentration was 10^5^ PFU/mL. The removal efficiency reached 2.5 log in 240 min with the initial concentration being 10^7^ PFU/mL. The results indicated that the initial pH did not impact the disinfection performance significantly. In the certain range, the removal efficiency increased with the increase in catalyst dosage, light intensity and temperature, but decreased with the increase in initial virus concentration. Free oxygen radicals have been shown to play a crucial role in phage f2 inactivation as well [202].

Materials with antiviral properties have a wide range of application. The ability to implement them into frequently touched surfaces may be a powerful tool in prevention of unwanted bacteriophages propagation and/or distribution.

**Table 3:** Methods of bacteriophages disinfection based on inorganic agents.

Agent	Bacteriophage	Remarks/Mechanism	References
AgNPs	T4, RG2014, MS2, PP7, ΦX174, SP6, 9NA	Electrostatic attraction, ROS-mediated oxidative damage, silver ions release	[178,179,185]
AgNPs-SiO_2_	MS2	Release of Ag^+^ ions	[183]
AuNPs	T1, T4, T7	Electrostatic attraction and hydrophobic interactions	[187]
TiO_2_ NPs	MS2	ROS-mediated oxidative damage	[203]
Fe/Ni NPs	F2	ROS-mediated oxidative damage	[188,189,190]
NP-CuO, NP-Ag-CuO	T4	Nucleophilic attack	[184,185]
nZVI	T4, T7, MS2, M13, F2, φX174	ROS-mediated oxidative damage, nucleic acids and capsid damage.	[172,188,189,190,191,192,204]
ZnOMgO, Cu_2_O CuO	M13, Qβ	Release of metal ions, particle adsorption, ROS-mediated oxidative damage	[165,205,206]
Cu_2_O/Al_2_O_3_ Cu/Al_2_O_3_	MS2	Copper ions release	[207]
IOCS—Iron oxide-coated sand	MS2, ΦX174	IOCS adsorption of bacteriophage	[168]
TiO_2_, CuO	T4, Qβ	Outer viral protein damage, bounding to TiO_2_ particles, ROS-mediated oxidative damage	[201,208]
Cu-TiO_2_	f2	ROS-mediated oxidative damage	[202]
Iron oxide	P22, MS2	Unknown	[169,171]
AgNO_3_, FeSO_4_, Al_2_(SO_4_)_3_, NiCl_2_, K_2_Cr_2_O_7_, CuSO_4_	Φ6, Qβ	Unknown	[165,195]
Cu_2_S CuI, CuCl	Qβ	Direct contact with the solid-state surface	[206]
SnCl_2_, SnCl_4_	T4	Inactivation most likely due to the presence of multiple sites of tin reactivity	[209]
Silver	ΦX174, MS2	Denaturation of MS2 Loss of capsid spikes in ΦX174	[164,166,210]
Ferrum	MS2, P22	Oxidants generated by iron oxidation, close contact, sorption of ferrous iron	[170,172]
Copper	Φ6, Φ8, PP7, ΦX174, PM2. MS2	ROS-mediated oxidative damage	[202]
Cu_2_NiSiCr, Cu_15_Ni_8_Sn Co_28_Cr_6_Mo	Φ6	Unknown	[195,196]
Ozone	MS2, ΦX174, Φ6, T7	Unknown	[161]
CO_2_	MS2, Qβ, T4	Capsid damage—CO_2_ uptake at high pressure and bursting of virions by depressurization	[162,163]

## 4. Organic Chemicals and Antimicrobials toward Bacteriophage Propagation Control

### 4.1. Disinfectants

Disinfectants based on organic compounds (Table 4) are commonly used to eradicate viruses and microorganisms from a variety of surfaces [211,212]. Among them, the best-known group are alcohols such as ethanol and isopropanol, characterized by relatively low toxicity and broad commercial distribution. With respect to the mechanism of action of ethanol, a study conducted by Maillard et al. showed capsid alterations on F116, a phage infecting *Pseudomonas aeruginosa* [213]. However, Halfhide [214] reported that 75% ethanol inactivated *Myoviridae* to levels below the detection limit, but did not cause more than a log reduction in *Siphoviridae*, highlighting the variability in ethanol’s efficacy against phages (taxa of this family no longer exist in phage nomenclature; species now belong to class *Caudoviricetes*). These observations clearly illustrate that it is imprudent to predict the efficacy of ethanol as a disinfectant against a specific phage.

Quaternary Ammonium Compounds (QACs) are popular sanitizers that can be used on certain food contact surfaces at home and industry due to their low toxicity. Cetyltrimethylammonium bromide (CTAB) is a class of aliphatic quaternary ammonium compounds which have a strong antimicrobial activity [215,216]. Study by Sands [216] showed a virucidal potency of CTAB against several bacteriophages (PM2, φ6, T4, PR4). The 3-log reduction of 10^6^ PFU/mL lysates was obtained after 15-minute incubation in 37°, but was dependent on both the compound concentration and phage type. The PM2 and φ6 phages were more sensitive to treatment than T4 and PR4. The more recent evaluation of CTAB activity was conducted by Ly-Chatain and colleagues targeting *Lactococcus* phage P001 (c2) associated with contaminations in the dairy industry [215]. After 1 min of contact with 0.125 mM CTAB, the c2 population was reduced from 6 to 1.5 log PFU/mL, and at 1 mM, concentration of CTAB phages were undetectable. However, the potency of CTAB was impaired in acidic pH and with an increased ionic strength of the medium. The authors explained this observation by the electrostatic interactions between cationic compounds and negatively charged particles such as bacteriophages or other compounds in a matrix. Activity of benzalkonium chloride-based QAC against 8 dairy phages infecting *L. lactis* (CB13, AF6, P1532, P001), *Lactobacillus* (B1) and streptococcal strains (2972) was also studied. The sanitizer potency was determined from 3 log to 6 log reduction within 15 min [212]. Moreover, this examination was conducted in the presence of 1% milk to mimic the dairy processing conditions.

Aldehydes such as glutaraldehyde or formaldehyde have been extensively used as disinfectants because of their broad spectrum of bactericidal, virucidal, fungicidal and sporicidal activity. Their biocide activity is based on the alkylation of hydroxyl, carbonyl and amino groups which affects DNA, RNA and protein synthesis. Moreover, glutaraldehyde is routinely used as a cross-linker due to its amine-binding ability. Maillard et al. [217] tested an efficiency of glutaraldehyde against MS2 and K coliphages and showed 4.1 to 5.2 log reduction of 10^8^ PFU/mL lysates (at 20 min) treated with 1% and 0.5% mixture respectively. It was in line with Jette et al. observations which demonstrated activity of glutaraldehyde-based disinfectants against phage f2 with 4 log particles reduction after 5 min and over 8-log reduction after 40 min [218].

### 4.2. Phytochemicals

Phytochemicals are a diverse group of naturally occurring chemical compounds found in plants [219,220]. These compounds have a wide range of biological properties, including antimicrobial and virucidal activities. The mechanisms of action of phytoncides vary, but they have been shown to disrupt the cell membrane, inhibit protein synthesis, modulate gene expression, and disrupt DNA replication, ultimately leading to the death of the microorganism. Overall, phytochemicals have significant potential as natural alternatives to synthetic disinfectants and antimicrobial agents [219,221]. The diversity of mechanisms of their action is presented in Table 4.

#### 4.2.1. Phenolic Compounds

Virucidal disinfectants based on phenolic compounds present variable impact on phage development [222,223]. Phenol and its derivatives have antifungal and antiviral properties. The breakdown of the plasma membrane, which allows the leakage of intracellular substances, is thought to be responsible for their antimicrobial effects. It was showed in study by Morita [223] that various phages were inactivated by polyphenols in the presence of cupric ion. The sensitivity of each phage to polyphenols was different. The T-odd series of phages were rapidly and efficiently inactivated by pyrocatechol at 37 °C. Phages fd and φX174 were less sensitive to pyrocatechol than the T-odd series. The results implicated that that free radicals of polyphenols and hydrogen peroxide are involved in phage inactivation. On the other hand, Maillard and colleagues showed that phenol has a moderate effect on the transduction of *P. aeruginosa* PAO by bacteriophage F116, has no effect on phage DNA within the capsid and no effect on various phage strand proteins unless the treatment lasts 20 min or longer [222].

A different perspective on the antiviral activity of polyphenols is presented by studies where plant secondary metabolites were employed. In a cross-study, Philippe and colleagues evaluated natural polyphenols activity (namely, quercetin, myricetin, p-coumaric acid, cinnamic acid, and kaempferol) against Vinitor162 and OE33PA bacteriophages of lactic acid bacterium *Oenococcus oeni* [224]. Seven polyphenols identified in their study inhibited the lytic propagation of OE33PA by an interference with its adsorption to the host cell. In contrast, any of the compounds showed activity in the presence of the distinct phage Vinitor162. In untreated cultures, Vinitor162 could lyse *O. oeni* after 20 h of incubation. Thus, the authors hypothesized that activity of polyphenolics is most likely related to phage OE33PA membrane receptor p2 block by the tested compounds [224]. This was further supported by a molecular docking analysis. Silva-Beltrán et al. in their study elucidated effects of tomato byproducts rich in polyphenolic agents (gallic, caffeic acids and quercetin) against *E. coli* bacteriophages MS2 and Av-5 [225]. Extracts showed an ability to reduce phage titer down to 6 logs [225]. Polyphenols exhibit activity to bind proteins, thereby forming protein-phenol aggregates [226]. More importantly, some agents were shown to impair the phage’s life cycle within its host. Such examples of specific activity have been demonstrated for representatives of flavones (e.g., quercetin, myricetin, and epigallocatechin) where arrest of DNA polymerase activity was observed [227]. Moreover, Yang and colleagues in their recent work showed epigallocatechin gallate impact on SOS response repression in *E. coli* resulting in the arrest of the development of phage 933W [228]. Catechins, also flavone compounds, were considered as antiviral agents in manufacturing cleaning wipes and filters as they showed biocide activity against T4 and T7 phage [229]. It was in line with mentioned above study by Morita of pyrocatechol virucidal potential [223]. Polyphenolic compounds showed wide range of antiviral activity [230,231] among them flavonoids emerge as the most promising agents for modulating bacteriophage development within its host. However, the full nature of the interactions of polyphenols with regard to their structure is still not fully evaluated. In particular, the basis of interaction with the virion particles or the effect on the stages of bacteriophage development remains to be elucidated. As presented above, catechins were able to affect phages in both ways depending on the particular strain.

#### 4.2.2. Isothiocyanates

Isothiocyanates (ITCs) are a most prominent group of bio-active compounds synthesized as a breakdown product of glucosinolates—sulfur rich phytochemicals originated from *Brassicaceae* plant family. ITCs are recently a subject of extensive studies due to their broad health benefits such as anticancer, anti-inflammatory, neuroprotective as well as antiviral and antibacterial potential [232,233,234,235,236,237]. The evaluation carried by our group revealed a potent activity of ITCs to impair the development of lambdoid *E. coli* phages [238,239]. This phenomenon is related to ability of ITCs to trigger a bacterial stress response (called a stringent response) mediated by small nucleotide alarmones (p)ppGpp [240]. (p)ppGpp molecules affect bacterial mechanisms of metabolism adaptation in response to environmental stresses such as nutrients deprivation. We showed that in the presence of ITCs (namely, sulforaphane, phenethyl-, allyl-, and benzyl-isothiocyanate), *E. coli* cells behave like under amino acid starvation conditions responding with elevated level of alarmone synthesis [238,239]. In general, the host cell starvation is an effective method to disrupt coliphages lytic development as demonstrated in comparative study by Los and colleagues [241]. However, Potrykus et al. precisely showed that induction of stringent response affects the expression from phage λ promoters which has consequences in virus progeny [242]. It was in line with our study describing (p)ppGpp negative impact on development of other lambdoid phages 933W and φ24B [243]. Moreover, the activity of ITCs not only results in inhibition of virus propagation but in that particular case also impairs the virulence of Shiga-toxigenic *E. coli,* which pathogenicity is related to bacteriophage development.

#### 4.2.3. Plant Extracts

Fruit extracts have been shown to be effective disinfectant agents against bacteriophages due to their natural origin and high concentration of phytochemicals. These phytochemicals, such as phenolic compounds and tannins, have been demonstrated to exhibit antimicrobial activity against a wide range of bacteria and viruses. In addition, the combination of these phytochemicals within an extract can often exhibit a synergistic effect, resulting in an even greater antimicrobial activity. One of the main advantages of using fruit extracts as disinfectants is their broad-spectrum activity, meaning they are effective against a wide range of microorganisms. This is particularly useful in the control of bacteriophages, which are difficult to eliminate due to their ability to infect and multiply within host cells. Furthermore, the use of fruit extracts as disinfectants is environmentally friendly, as they are derived from natural sources and do not produce harmful byproducts during the disinfection process, thus making them a good alternative to chemical agents.

Pomegranate extracts are known for their antimicrobial potential [244,245]. These effects are attributed to its high content of polyphenols, including mainly hydrolysable tannins (ellagitannins), such as punicalagin isomers, with small amounts of ellagic acid and anthocyanins (delphinidin, cyanidins, and pelargonidin) and their glycosides. Su et al. demonstrated a virucidal activity of pomegranate juice and pomegranate polyphenolic (PP) on MS2 phage [221,246,247]. Their activity was dependent on initial phage titer as well as concentration of PP. Thus, MS2 at low initial titers (10^5^) was reduced by 0.41, 0.45, and 0.93 log PFU/mL and at high initial titers (10^8^) by 0.32, 0.41, and 0.72 log PFU/mL after 4, 8, and 16 mg/mL of PP treatment, respectively. Moreover, Stewart and colleagues employed the pomegranate extract to inactivate bacteriophages in assay aimed to detection of specific bacterial pathogens [248]. A crucial step for this assay was deactivation of viruses inside a bacterium using pomegranate rind extract (PRE) with no harm to bacterial culture. In combination with ferrous sulphate, PRE can provide about an 11-log reduction in phage titer within 3 min, and it activity has been shown for a range of bacterial hosts including *P. aeruginosa*, *Salmonella typhimurium,* and *S. aureus* (NCIMB 10116, Felix O-1, and NCIMB 9563, respectively) [248]. The mode of action of high PP concentration in extracts are considered to be a capsid denaturation as demonstrated in other study, according to TEM visualization [249]. Overall, the obtained results showed that grape seeds and pomegranate extracts are significant inhibitory agents for phages. There are a number of publications identifying the inhibitory activity of these components on bacteriophages. In one of these studies, it was reported that cranberry juice, grape juice and orange juice had an inhibitory effect on bacteriophage T2 [250]. Study by Su et al. from 2010, demonstrated that different concentrations of cranberry juice and cranberry proanthocyanidins were found to reduce titers of MS2 and ΦX174 bacteriophages [251]. Similarly, it was found that potato peel extract had an inhibitory effect on Av-05 and MS2 bacteriophages [252].

### 4.3. Antibiotics

The antimicrobial agents serve for decades to control bacterial and fungal infections. In fact, most of drugs were found to be produced by microorganisms as secondary metabolites. In the natural environment, they play an important role in the mechanism of microbes’ self-protection, as well as competition for habitats. Some of these small molecules can also act as potent inhibitors of phage replication and represent a widespread anti-phage defense system [253,254,255]. Although the antibiotic treatment of bacterial cultures can affect their development, the concentrations of drugs used in presented below studies did not affect the host development or in some cases the resistant strains were used.

Among antibiotics, in particular, a group of compounds produced by *Streptomyces*, the aminoglycosides, have proven to be highly effective in controlling bacteriophages [256]. Aminoglycosides have bactericidal potential, and their mode of action is related to a binding affinity for nucleic acids. In bacteria, their target is the 30S subunit of the ribosomes, resulting in disruption of protein biosynthesis due to translation blockage or mistranslation events. In early 1960s, Brock and colleagues [257,258] showed the streptomycin can inhibit development of *E. coli* MS2 phage and certain streptococcal bacteriophages. According to their findings all of the DNA viruses tested were resistant, but the RNA virus was sensitive to the drug. At the time, it was claimed that streptomycin inhibited both the adsorption/injection phase and replication of viral genetic material. Recently, study by Jiang shed new light to aminoglycoside anti-phage action [259]. The authors showed that presence of kanamycin, hygromycin, or streptomycin leads to inhibition of the DNA replication of mycobacteriophages. They employed natural phage D29 and engineered phAE159 to comprehensive evaluation of aminoglycosides action. However, Jiang and colleagues also tested these drugs’ activity using *E. coli* DNA phages T7 and λ, and showed no effect to these phages. Thus, the authors hypothesized that amino sugar group of aminoglycosides might selectively inhibit mycobacteriophage DNA replication. These findings are in line with another elegant work by Kever et al. [260] where, using bacterial hosts expressing aminoglycoside resistance plasmid cassettes, aminoglycosides are demonstrated to present wide anti-phage properties. Activity of aminoglycosides was proved by employing viruses of Gram-negative *E. coli*, as well as Gram-positive bacteria such as *Corynebacterium glutamicum* and *Streptomyces venezuelae*. The study revealed that phage DNA was detected inside cells in the presence of aminoglycosides. Together with the observation that amplification of phage DNA was strongly impaired, these results suggest that the blockage exerted by aminoglycosides occurs mostly after DNA injection but before genome replication.

Peptide antibiotics is a group of antimicrobial and cytostatic substances with a highly diverse structure and mechanisms of action. Nonetheless, some glycopeptide drugs, namely, phleomycin and bleomycin were shown to effectively affect virus propagation through genetic material alteration. Watanabe and August observed activity of phleomycin against both DNA and RNA phages of *E. coli* [261]. This drug shows specific affinity to single and double strand RNA resulting in impairment of T2 and R23 phages development according to the study. Moreover, inhibition by phleomycin of viral RNA polymerase and DNA-dependent RNA polymerase was proved by in vitro evaluation. Similarly, Post and Price revealed that phleomycin acts also as an effective inhibitor of the replication of *Bacillus subtilis* bacteriophage PBS2 [262]. In their study, phage DNA synthesis was severely inhibited by drug presence, thereby blocking the synthesis of late virion proteins. Another drug, bleomycin, is considered as a DNA binding agent and thus affects viruses’ activity. Cloos and colleagues showed the drug potential to damage PM2 phage genome [263]. Bleomycin mediates inner cross-links in phage PM2 DNA. The cross-links are observed only when the reactant covalently closed circular duplex DNA contains either positive or negative superhelical turns. However, due to its abilities to cause alterations in DNA structure bleomycin is also a potent prophage inducer via SOS response activation [264]. One of the other examples of polypeptide drug that shows abilities to bind to DNA is quinomycin A. Quinomycin A is a compound with circular structure with potent antibacterial, anticancer and antiviral activities. The drug was shown to inhibit the T2 phage development without measurable interference with the synthesis of the phage DNA, RNA, or protein [265]. Therefore, the authors assumed that quinomycin A inhibits phage development at a step during maturation, possibly in association of DNA and head protein.

**Table 4:** The organic agents used for phage control.

Group	Agent	Bacteriophage	Remarks/Mechanism	References
Disinfectants	Ethanol, isopropanol	MS2, K, CYM, 021-4, 021-5, 0BJ, F116	Capsid denaturation	[211,217,222]
	Glutaraldehyde	F2, MS2	Alkylation of nucleic acid	[217,218]
	octenidine dihydrochloride	F2, MS2, Entb_43, Entb_45	Unknown	[266,267]
	Peracetic acid	F116, Ib_3_	Capsid denaturation	[211,268]
	Quaternary ammonium	MS2, c2, P008, CB13, AF6, P1532, λ		[212,215,216,253]
	Phenol	F116, φX174	Genome damage, ROS	[222,223,268]
Phytochemicals	Caffeic acid	Av-5, MS2, φX174	Inhibition of replication	[223,225]
	Gallic acid	Av-5, MS2, PL-1, φX174	Inhibition of replication and infection	[223,225,226,230]
	Carvacrol	933 W, MS2	Inhibition of enzymatic activity of host proteins	[269,270]
	Tannic acid	λ, MS2	ROS, capsid denaturation	[231]
	Epigallocatechin gallate	933 J	Repression of *SOS* response and phage gene expression	[228]
	Catechin	T4D, T7	Structural damage, modulation of gene expression	[227,229]
	Cinnamaldehyde	933 W	Repression of *recA*	[271,272]
	Thymol	φC, φEC	Unknown	[273]
	Chitosan	MS2, φX174, 1–97 A, c2, 933W	Capsid denaturation	[215,274,275,276]
	Quercetin, myricetin, p-coumaric acid, cinnamic acid, kaempferol	OE33PA	Inhibition of phage adsorption	[224]
	Isothiocyanates	933W, φ24B, λ	Stringent response induction	[238,239]
	Tea extracts	Felix O-1, P22	Unknown	[277]
	Pomegranate juice	MS2, S. aureus phage PHAGESTAPH, Felix O-1, NCIMB 9563	Capsid denaturation	[221,245,246,247,248]
	Cranberry juice	T2, T4, MS2, φX174	Capsid denaturation, adsorption prevention	[250,251,278]
	Propolis	MS2, Av-08	Capsid denaturation, adsorption and internalization prevention	[279,280,281]
	Ascorbic acid	δA, φX174, T7, P22, D29, PM2, MS2	Genome damage	[282,283,284]
Antibiotics	Bleomycin	PM2	DNA damage	[263]
	Apramycin	λ, Alderaan	Replication impairment	[260]
	Streptomycin	f2, MS2	Block of genetic material injection step	[257,258]
	Rugulosin	MS2, GA and δβ	Early steps of development impairing, RNA injection block	[255]
	Kanamycin, hygromycin, streptomycin	phAE159, D29		[259]
	Quinomycin A	T2	Block of association of DNA and head protein	[265]
	Phleomycin	R23, T2 PBS2	Inhibition of phage-specific RNA synthesis	[261,262]
	Nalidixic acid	PBS2	Inhibition of infection	[285]

## 5. Perspectives

To our best knowledge, to date, there is no systematic approach to study the bacteriophages disinfection methods and spread monitoring. Therefore, we recognize the urgent need to develop common standard methods to reduce future risks related to the widespread use of phages. Our remarks under perspective of standardization procedures and good practice in phage usage are as follows:

- The relevant methods of phage eradication should take into account the differences in the structure and virus type.
- The research aimed at developing new methods of phage infection prevention should include not only establishing of novel compounds but also their utilization.
- The standards of disinfection effectiveness should be increased from typically 3-log decrease in the phage titer to 6 log (due to the need to reduce a higher relative titer of viral particles vs. bacterial cells).
- The phage usage in medicine and biotechnology should be under strict monitoring to prevent from uncontrolled spreading in the environment, which is especially important for phages imported from distant ecosystems.
- It should be taken into consideration to establish the rules of monitoring bacteriophage genetic variations and diversity to maintain the safety of phage use, especially in clinical practice.

## 6. Conclusions

The potential of bacterial viruses, bacteriophages, nowadays gains a wide attention due to their role as useful tools in many fields. However, their uncontrolled distribution by their use in medicine, food production, and preservation as well as in bio-technology poses a potential serious risk, which should not be underestimated. As the main vector of horizontal gene transfer and driver of microbial variability, bacteriophages can become a trigger for threats to humans. Especially, the use of bioengineered strains of phages implicates potential risks. The ubiquity of bacteriophages and their persistence in the environment raise concern about their involvement in antimicrobial resistance genes and/or virulence factors transmission among different biomes and the generation of multi-resistant pathogenic bacteria. Thus, the more common use of bacteriophages in medicine and biotechnology should be preceded by research aimed in clear understanding of phage–phage and phage–bacterium dynamics as well. Thus, efforts aimed at establishing instruments to control the development and monitor the spread of bacteriophages should go simultaneously with their widespread use. The development of relevant antiviral agents and methods of phage eradication is an indispensable and necessary element of modern biotechnology and clinical practice. Moreover, the vast part of methods for bacteriophage infection prevention could be either the same or at least combined with already known and used methods for disinfection and bacterial pathogens eradication. Thus, the better understanding of the mechanisms of disinfectant actions and effects is a key step in establishing the trustful methods for microorganism control.

## Figures and Tables

**Table 1:** Diversity of bacteriophages in accordance with taxonomic classification.

Phylum	Class	Order	Family	Details	Genome	Phages	Structure
Uroviricota	Caudoviricetes			Contractile tail	dsDNA (L)	T2, T4, T6, P1, K, PBS2, Entb_43	With tail
				Non-contractile tail		λ, T1, T5, HK97, 933W, Φ24B, C-36, P680, P22	
			Autographiviridae	Encode their own single subunit RNA polymerase		T7, T3, SP6	
Phixviricota	Malgrandaviricetes	Petitvirales	Microviridae	Non-enveloped, round	ssDNA (C)	ΦX174	Polyhedral
Preplasmiviricota	Tectiliviricetes	Linavirales	Corticoviridae	Complex capsid, lipids	dsDNA (C, S)	PM2	
		Kalamavirales	Tectiviridae	Double capsid, lipids, pseudo-tail	dsDNA (L)	PRD1	
		Belfryvirales	Turriviridae	Icosahedron with protrude protein turrets	dsDNA (C)	STIV	
Dividoviricota	Laserviricetes	Halopanivirales	Sphaerolipoviridae	Double capsid, lipids	dsDNA (L)	SH1	
Lenarviricota	Leviviricetes		Fiersviridae	Like poliovirus	ssRNA (L)	MS2, PP7, R17, R23, f2, Qβ	
Duplornaviricota	Vidaverviricetes	Mindivirales	Cystoviridae	Envelope, lipids	dsRNA (L, M)	Φ6	
Hofneiviricota	Faserviricetes	Tubulavirales	Inoviridae	Long filamentous, short stem	ssDNA (C)	M13, SJC3, δA	Filamentous
Taleaviricota	Tokiviricetes	Ligamenvirales	Ungulaviridae	Envelope, lipids	dsDNA (L)	AFV1	
			Rudiviridae	Rigid rods type, TMV	dsDNA (L)	SIRV-2	
			Plasmaviridae	Envelope, without lipid capsid	dsDNA (C, S)	L2	Pleomorphic
			Fuselloviridae	Lemon shape, envelope	dsDNA (C, S)	SSV1, ASV1	
			Halspiviridae	Spindle-shaped, envelope	dsDNA (L, S)	His1	
			Guttaviridae	Drop shape	dsDNA (C, S)	APOV1	
			Ampullaviridae	Bottle shape, NC helical	dsDNA (L)	ABV	
			Portogloboviridae	Icosahedral, outer protein shell, inner lipid layer	dsDNA (C)	SPV1	Icosahedral

## Data Availability

Not applicable.

## References

[1] Ofir G., Sorek R. (2018). Contemporary Phage Biology: From Classic Models to New Insights. Cell.

[2] Shapiro O.H., Kushmaro A. (2011). Bacteriophage Ecology in Environmental Biotechnology Processes. Curr. Opin. Biotechnol..

[3] Santiago-Rodriguez T.M., Hollister E.B. (2019). Human Virome and Disease: High-Throughput Sequencing for Virus Discovery, Identification of Phage-Bacteria Dysbiosis and Development of Therapeutic Approaches with Emphasis on the Human Gut. Viruses.

[4] Al-Hindi R.R., Teklemariam A.D., Alharbi M.G., Alotibi I., Azhari S.A., Qadri I., Alamri T., Harakeh S., Applegate B.M., Bhunia A.K. (2022). Bacteriophage-Based Biosensors: A Platform for Detection of Foodborne Bacterial Pathogens from Food and Environment. Biosensors.

[5] Chen Q., Dharmaraj T., Cai P.C., Burgener E.B., Haddock N.L., Spakowitz A.J., Bollyky P.L. (2022). Bacteriophage and Bacterial Susceptibility, Resistance, and Tolerance to Antibiotics. Pharmaceutics.

[6] Fernández L., Escobedo S., Gutiérrez D., Portilla S., Martínez B., García P., Rodríguez A. (2017). Bacteriophages in the Dairy Environment: From Enemies to Allies. Antibiotics.

[7] Briggiler Marcó M., Mercanti D.J. (2021). Bacteriophages in Dairy Plants. Adv. Food Nutr. Res..

[8] Simmonds P., Aiewsakun P. (2018). Virus Classification—Where Do You Draw the Line?. Arch. Virol..

[9] Sulakvelidze A., Alavidze Z., Morris J. (2001). Bacteriophage Therapy. Antimicrob. Agents Chemother..

[10] Loś J.M., Loś M., Wegrzyn G. (2011). Bacteriophages Carrying Shiga Toxin Genes: Genomic Variations, Detection and Potential Treatment of Pathogenic Bacteria. Future Microbiol..

[11] Düzgüneş N., Sessevmez M., Yildirim M. (2021). Bacteriophage Therapy of Bacterial Infections: The Rediscovered Frontier. Pharmaceuticals.

[12] Lin J., Du F., Long M., Li P. (2022). Limitations of Phage Therapy and Corresponding Optimization Strategies: A Review. Molecules.

[13] Bao Q., Li X., Han G., Zhu Y., Mao C., Yang M. (2019). Phage-Based Vaccines. Adv. Drug Deliv. Rev..

[14] Nagano K., Tsutsumi Y. (2021). Phage Display Technology as a Powerful Platform for Antibody Drug Discovery. Viruses.

[15] Harada L.K., Silva E.C., Campos W.F., del Fiol F.S., Vila M., Dąbrowska K., Krylov V.N., Balcão V.M. (2018). Biotechnological Applications of Bacteriophages: State of the Art. Microbiol. Res..

[16] Colavecchio A., Cadieux B., Lo A., Goodridge L.D. (2017). Bacteriophages Contribute to the Spread of Antibiotic Resistance Genes among Foodborne Pathogens of the Enterobacteriaceae Family—A Review. Front. Microbiol..

[17] Jebri S., Rahmani F., Hmaied F. (2021). Bacteriophages as Antibiotic Resistance Genes Carriers in Agro-Food Systems. J. Appl. Microbiol..

[18] Chiang Y.N., Penadés J.R., Chen J. (2019). Genetic Transduction by Phages and Chromosomal Islands: The New and Noncanonical. PLoS Pathog..

[19] Penadés J.R., Chen J., Quiles-Puchalt N., Carpena N., Novick R.P. (2015). Bacteriophage-Mediated Spread of Bacterial Virulence Genes. Curr. Opin. Microbiol..

[20] Boyd E.F. (2012). Bacteriophage-Encoded Bacterial Virulence Factors and Phage-Pathogenicity Island Interactions. Adv. Virus Res..

[21] Castillo D., Kauffman K., Hussain F., Kalatzis P., Rørbo N., Polz M.F., Middelboe M. (2018). Widespread Distribution of Prophage-Encoded Virulence Factors in Marine Vibrio Communities. Sci. Rep..

[22] Smith N.L., Taylor E.J., Lindsay A.M., Charnock S.J., Turkenburg J.P., Dodson E.J., Davies G.J., Black G.W. (2005). Structure of a Group A Streptococcal Phage-Encoded Virulence Factor Reveals a Catalytically Active Triple-Stranded β-Helix. Proc. Natl. Acad. Sci. USA.

[23] Zhang Y., Guo Y., Qiu T., Gao M., Wang X. (2022). Bacteriophages: Underestimated Vehicles of Antibiotic Resistance Genes in the Soil. Front. Microbiol..

[24] Alavidze Z., Aminov R., Betts A., Bardiau M., Bretaudeau L., Caplin J., Chanishvili N., Coffey A., Cooper I., de Vos D. (2016). Silk Route to the Acceptance and Re-Implementation of Bacteriophage Therapy. Biotechnol. J..

[25] Pirnay J.P., Blasdel B.G., Bretaudeau L., Buckling A., Chanishvili N., Clark J.R., Corte-Real S., Debarbieux L., Dublanchet A., de Vos D. (2015). Quality and Safety Requirements for Sustainable Phage Therapy Products. Pharm. Res..

[26] Bertrand I., Schijven J.F., Sánchez G., Wyn-Jones P., Ottoson J., Morin T., Muscillo M., Verani M., Nasser A., de Roda Husman A.M. (2012). The Impact of Temperature on the Inactivation of Enteric Viruses in Food and Water: A Review. J. Appl. Microbiol..

[27] Müller-Merbach M., Neve H., Hinrichs J. (2005). Kinetics of the Thermal Inactivation of the *Lactococcus lactis* Bacteriophage P008. J. Dairy Res..

[28] Şanlibaba P., Buzrul S., Akkoç N., Alpas H., Akçlik M. (2009). Thermal Inactivation Kinetics of *Lactococcus lactis* Subsp. Lactis Bacteriophage Pll98-22. Acta Biol. Hung..

[29] Sadat Hosseini S.R., Edalatian Dovom M.R., Yavarmanesh M., Abbaszadegan M. (2017). Thermal Inactivation of MS2 Bacteriophage as a Surrogate of Enteric Viruses in Cow Milk. J. Verbrauch. Lebensm..

[30] García Fontán M.C., Martínez S., Franco I., Carballo J. (2006). Microbiological and Chemical Changes during the Manufacture of Kefir Made from Cows’ Milk, Using a Commercial Starter Culture. Int. Dairy J..

[31] Atamer Z., Hinrichs J. (2010). Thermal Inactivation of the Heat-Resistant *Lactococcus lactis* Bacteriophage P680 in Modern Cheese Processing. Int. Dairy J..

[32] Buzrul S., Öztürk P., Alpas H., Akcelik M. (2007). Thermal and Chemical Inactivation of Lactococcal Bacteriophages. LWT Food Sci. Technol..

[33] Atamer Z., Dietrich J., Müller-Merbach M., Neve H., Heller K.J., Hinrichs J. (2009). Screening for and Characterization of *Lactococcus lactis* Bacteriophages with High Thermal Resistance. Int. Dairy J..

[34] Madera C., Monjardín C., Suárez J.E. (2004). Milk Contamination and Resistance to Processing Conditions Determine the Fate of *Lactococcus lactis* Bacteriophages in Dairies. Appl. Environ. Microbiol..

[35] Wagner N., Samtlebe M., Franz C.M.A.P., Neve H., Heller K.J., Hinrichs J., Atamer Z. (2017). Dairy Bacteriophages Isolated from Whey Powder: Thermal Inactivation and Kinetic Characterisation. Int. Dairy J..

[36] Wagner N., Matzen S., Walte H.G., Neve H., Franz C.M.A.P., Heller K.J., Hammer P. (2018). Extreme Thermal Stability of *Lactococcus lactis* Bacteriophages: Evaluation of Phage Inactivation in a Pilot-Plant Pasteurizer. LWT.

[37] Charles K.J., Shore J., Sellwood J., Laverick M., Hart A., Pedley S. (2009). Assessment of the Stability of Human Viruses and Coliphage in Groundwater by PCR and Infectivity Methods. J. Appl. Microbiol..

[38] Blazanin M., Lam W.T., Vasen E., Chan B.K., Turner P.E. (2022). Decay and Damage of Therapeutic Phage OMKO1 by Environmental Stressors. PLoS ONE.

[39] Qiu X. (2012). Heat Induced Capsid Disassembly and DNA Release of Bacteriophage λ. PLoS ONE.

[40] Duda R.L., Ross P.D., Cheng N., Firek B.A., Hendrix R.W., Conway J.F., Steven A.C. (2009). Structure and Energetics of Encapsidated DNA in Bacteriophage HK97 Studied by Scanning Calorimetry and Cryo-Electron Microscopy. J. Mol. Biol..

[41] Sanchis A.G. (2021). Thermal Inactivation of Viruses and Bacteria with Hot Air Bubbles in Different Electrolyte Solutions. Substantia.

[42] Caldeira J.C., Peabody D.S. (2007). Stability and Assembly in Vitro of Bacteriophage PP7 Virus-like Particles. J. Nanobiotechnol..

[43] Cele N. (2009). Strategies to Control Bacteriophage Infection in a Threonine Bioprocess. http://hdl.handle.net/10321/451.

[44] Hazem A. (2002). Effects of Temperatures, PH-Values, Ultra-Violet Light, Ethanol and Chloroform on the Growth of Isolated Thermophilic Bacillus Phages. New Microbiol..

[45] Baines B. (2005). A Comparison of the Effects of Microwave Irradiation and Heat Treatment of T4 and T7 Bacteriophage. J. Exp. Microbiol. Immunol..

[46] Atamer Z., Dietrich J., Neve H., Heller K.J., Hinrichs J. (2010). Influence of the Suspension Media on the Thermal Treatment of Mesophilic Lactococcal Bacteriophages. Int. Dairy J..

[47] Chopin M.C. (1980). Resistance of 17 Mesophilic Lactic Streptococcus Bacteriophages to Pasteurization and Spray-Drying. J. Dairy Res..

[48] Kowalski W. (2009). Ultraviolet Germicidal Irradiation Handbook: UVGI for Air and Surface Disinfection. Ultraviolet Germicidal Irradiation Handbook: UVGI for Air and Surface Disinfection.

[49] Qureshi Z., Yassin M. (2013). Role of Ultraviolet (UV) Disinfection in Infection Control and Environmental Cleaning. Infect. Disord. Drug Targets.

[50] Tseng C.C., Li C.S. (2007). Inactivation of Virus-Containing Aerosols by Ultraviolet Germicidal Irradiation. Aerosol Sci. Technol..

[51] Rauth A.M. (1965). The Physical State of Viral Nucleic Acid and the Sensitivity of Viruses to Ultraviolet Light. Biophys. J..

[52] Battigelli D.A., Sobsey M.D., Lobe D.C. (1993). The Inactivation of Hepatitis a Virus and Other Model Viruses by UV Irradiation. Water Sci. Technol..

[53] Nuanualsuwan S., Mariam T., Himathongkham S., Cliver D.O. (2002). Ultraviolet Inactivation of Feline Calicivirus, Human EntericViruses and Coliphages¶. Photochem. Photobiol..

[54] Kim D.K., Kim S.J., Kang D.H. (2017). Inactivation Modeling of Human Enteric Virus Surrogates, MS2, Qβ, and ΦX174, in Water Using UVC-LEDs, a Novel Disinfecting System. Food Res. Int..

[55] Rodriguez R.A., Bounty S., Beck S., Chan C., McGuire C., Linden K.G. (2014). Photoreactivation of Bacteriophages after UV Disinfection: Role of Genome Structure and Impacts of UV Source. Water Res..

[56] Aoyagi Y., Takeuchi M., Yoshida K., Kurouchi M., Yasui N., Kamiko N., Araki T., Nanishi Y. (2011). Inactivation of Bacterial Viruses in Water Using Deep Ultraviolet Semiconductor Light-Emitting Diode. J. Environ. Eng..

[57] Bartolomeu M., Braz M., Costa P., Duarte J., Pereira C., Almeida A. (2022). Evaluation of UV-C Radiation Efficiency in the Decontamination of Inanimate Surfaces and Personal Protective Equipment Contaminated with Phage Φ6. Microorganisms.

[58] Meng Q.S., Gerba C.P. (1996). Comparative Inactivation of Enteric Adenoviruses, Poliovirus and Coliphages by Ultraviolet Irradiation. Water Res..

[59] Pinon A., Vialette M. (2018). Survival of Viruses in Water. Intervirology.

[60] Vatter P., Hoenes K., Hessling M. (2021). Blue Light Inactivation of the Enveloped RNA Virus Phi6. BMC Res. Notes.

[61] Tomb R.M., Maclean M., Herron P.R., Hoskisson P.A., MacGregor S.J., Anderson J.G. (2014). Inactivation of Streptomyces Phage ΦC31 by 405 Nm Light: Requirement for Exogenous Photosensitizers?. Bacteriophage.

[62] Vatter P., Hoenes K., Hessling M. (2021). Photoinactivation of the Coronavirus Surrogate Phi6 by Visible Light. Photochem. Photobiol..

[63] Chrysikopoulos C.v., Manariotis I.D., Syngouna V.I. (2013). Virus Inactivation by High Frequency Ultrasound in Combination with Visible Light. Colloids Surf. B Biointerfaces.

[64] van der Walt E., Grundling M. (2004). The Use of Ultraviolet Light Alone, or in Combination with Cavitational Flow and Ultrasonic Devices, to Inactivate Protozoan Cysts and Oocysts in the Small and Large Scale Treatment of Drinking Water Final Report. https://www.wrc.org.za/wp-content/uploads/mdocs/K5-1224%20f1.pdf.

[65] Fang J., Liu H., Shang C., Zeng M., Ni M., Liu W. (2013). *E. coli* and Bacteriophage MS2 Disinfection by UV, Ozone and the Combined UV and Ozone Processes. Front. Environ. Sci. Eng..

[66] Kim J.Y., Lee C., Cho M., Yoon J. (2008). Enhanced Inactivation of *E. coli* and MS-2 Phage by Silver Ions Combined with UV-A and Visible Light Irradiation. Water Res..

[67] Brauch G., Hansler U., Ludwia H. (2006). The Effect of Pressure on Bacteriophages. High Press. Sci. Technol..

[68] D’Souza D.H., Xiaowei S.U., Adrienne R., Harte F. (2009). High-Pressure Homogenization for the Inactivation of Human Enteric Virus Surrogates. J. Food Prot..

[69] Mercanti D.J., Guglielmotti D.M., Patrignani F., Reinheimer J.A., Quiberoni A. (2012). Resistance of Two Temperate Lactobacillus Paracasei Bacteriophages to High Pressure Homogenization, Thermal Treatments and Chemical Biocides of Industrial Application. Food Microbiol..

[70] Capra M.L., Patrignani F., del Lujan Quiberoni A., Reinheimer J.A., Lanciotti R., Guerzoni M.E. (2009). Effect of High Pressure Homogenization on Lactic Acid Bacteria Phages and Probiotic Bacteria Phages. Int. Dairy J..

[71] Müller-Merbach M., Rauscher T., Hinrichs J. (2005). Inactivation of Bacteriophages by Thermal and High-Pressure Treatment. Int. Dairy J..

[72] Moroni O., Jean J., Autret J., Fliss I. (2002). Inactivation of Lactococcal Bacteriophages in Liquid Media Using Dynamic High Pressure. Int. Dairy J..

[73] Solomon L., Zeegen P., Eiserling F.A. (1966). The Effects of High Hydrostatic Pressure on Coliphage T-4. Biochim. Biophys. Acta Biophys. Incl. Photosynth..

[74] Lin K., Schulte C.R., Marr L.C. (2020). Survival of MS2 and Φ6 Viruses in Droplets as a Function of Relative Humidity, PH, and Salt, Protein, and Surfactant Concentrations. PLoS ONE.

[75] Kormuth K.A., Lin K., Prussin A.J., Vejerano E.P., Tiwari A.J., Cox S.S., Myerburg M.M., Lakdawala S.S., Marr L.C. (2018). Influenza Virus Infectivity Is Retained in Aerosols and Droplets Independent of Relative Humidity. J. Infect. Dis..

[76] Prussin A.J., Schwake D.O., Lin K., Gallagher D.L., Buttling L., Marr L.C. (2018). Survival of the Enveloped Virus Phi6 in Droplets as a Function of Relative Humidity, Absolute Humidity, and Temperature. Appl. Environ. Microbiol..

[77] Songer J.R. (1967). Influence of Relative Humidity on the Survival of Some Airborne Viruses. Appl. Microbiol..

[78] Darimani H.S., Ito R., Funamizu N., Maiga A.H., Darimani H.S., Ito R., Funamizu N., Maiga A.H. (2018). Effect of Post-Treatment Conditions on the Inactivation of MS2 Bacteriophage as Indicator for Pathogenic Viruses after the Composting Process. J. Agric. Chem. Environ..

[79] Rockey N., Arts P.J., Li L., Harrison K.R., Langenfeld K., Fitzsimmons W.J., Lauring A.S., Love N.G., Kaye K.S., Raskin L. (2020). Humidity and Deposition Solution Play a Critical Role in Virus Inactivation by Heat Treatment of N95 Respirators. mSphere.

[80] Casanova L.M., Waka B. (2013). Survival of a Surrogate Virus on N95 Respirator Material. Infect. Control Hosp. Epidemiol..

[81] Lin K., Marr L.C. (2020). Humidity-Dependent Decay of Viruses, but Not Bacteria, in Aerosols and Droplets Follows Disinfection Kinetics. Environ. Sci. Technol..

[82] Ezugbe E.O., Rathilal S. (2020). Membrane Technologies in Wastewater Treatment: A Review. Membranes.

[83] Fane A.G., Wang R., Jia Y. (2011). Membrane Technology: Past, Present and Future. Membr. Desalin. Technol..

[84] Kang S., Pinault M., Pfefferle L.D., Elimelech M. (2007). Single-Walled Carbon Nanotubes Exhibit Strong Antimicrobial Activity. Langmuir.

[85] Al-Jumaili A., Alancherry S., Bazaka K., Jacob M.v. (2017). Review on the Antimicrobial Properties of Carbon Nanostructures. Materials.

[86] Li Q., Mahendra S., Lyon D.Y., Brunet L., Liga M.V., Li D., Alvarez P.J.J. (2008). Antimicrobial Nanomaterials for Water Disinfection and Microbial Control: Potential Applications and Implications. Water Res..

[87] Brady-Estévez A.S., Kang S., Elimelech M. (2008). A Single-Walled-Carbon-Nanotube Filter for Removal of Viral and Bacterial Pathogens. Small.

[88] Brady-Estévez A.S., Schnoor M.H., Vecitis C.D., Saleh N.B., Elimelech M. (2010). Multiwalled Carbon Nanotube Filter: Improving Viral Removal at Low Pressure. Langmuir.

[89] Brady-Estévez A.S., Schnoor M.H., Kang S., Elimelech M. (2010). SWNT-MWNT Hybrid Filter Attains High Viral Removal and Bacterial Inactivation. Langmuir.

[90] Brady-Estévez A.S., Nguyen T.H., Gutierrez L., Elimelech M. (2010). Impact of Solution Chemistry on Viral Removal by a Single-Walled Carbon Nanotube Filter. Water Res..

[91] Redman J.A., Grant S.B., Olson T.M., Adkins J.M., Jackson J.L., Castillo M.S., Yanko W.A. (1999). Physicochemical Mechanisms Responsible for the Filtration and Mobilization of a Filamentous Bacteriophage in Quartz Sand. Water Res..

[92] Tsen K.T., Tsen S.W.D., Chang C.L., Hung C.F., Wu T.C., Kiang J.G. (2007). Inactivation of Viruses with a Very Low Power Visible Femtosecond Laser. J. Phys. Condens. Matter.

[93] Tsen K.T., Tsen S.W.D., Chang C.L., Hung C.F., Wu T.C., Kiang J.G. (2007). Inactivation of Viruses by Coherent Excitations with a Low Power Visible Femtosecond Laser. Virol. J..

[94] Tsen K.-T., Tsen S.-W.D., Chang C.-L., Hung C.-F., Wu T.-C., Kiang J.G. (2007). Inactivation of Viruses by Laser-Driven Coherent Excitations via Impulsive Stimulated Raman Scattering Process. J. Biomed. Opt..

[95] Tsen K.-T., Tsen S.-W.D., Fu Q., Lindsay S.M., Kibler K., Jacobs B., Wu T.-C., Karanam B., Jagu S., Roden R.B.S. (2009). Photonic Approach to the Selective Inactivation of Viruses with a Near-Infrared Subpicosecond Fiber Laser. J. Biomed. Opt..

[96] Tsen S.W.D., Wu T.C., Kiang J.G., Tsen K.T. (2012). Prospects for a Novel Ultrashort Pulsed Laser Technology for Pathogen Inactivation. J. Biomed. Sci..

[97] Tsen S.W.D., Kingsley D.H., Poweleit C., Achilefu S., Soroka D.S., Wu T.C., Tsen K.T. (2014). Studies of Inactivation Mechanism of Non-Enveloped Icosahedral Virus by a Visible Ultrashort Pulsed Laser. Virol. J..

[98] Tsen K.T., Tsen S.-W.D., Fu Q., Lindsay S.M., Li Z., Cope S., Vaiana S., Kiang J.G. (2011). Studies of Inactivation of Encephalomyocarditis Virus, M13 Bacteriophage, and Salmonella Typhimurium by Using a Visible Femtosecond Laser: Insight into the Possible Inactivation Mechanisms. J. Biomed. Opt..

[99] Tsen S.W.D., Tsen Y.S.D., Tsen K.T., Wu T.C. (2010). Selective Inactivation of Viruses with Femtosecond Laser Pulses and Its Potential Use for *in Vitro* Therapy. J. Healthc. Eng..

[100] Berchtikou A., Greschner A.A., Tijssen P., Gauthier M.A., Ozaki T. (2020). Accelerated Inactivation of M13 Bacteriophage Using Millijoule Femtosecond Lasers. J. Biophotonics.

[101] Alshraiedeh N.H., Alkawareek M.Y., Gorman S.P., Graham W.G., Gilmore B.F. (2013). Atmospheric Pressure, Nonthermal Plasma Inactivation of MS2 Bacteriophage: Effect of Oxygen Concentration on Virucidal Activity. J. Appl. Microbiol..

[102] Venezia R.A., Orrico M., Houston E., Yin S.-M., Naumova Y.Y. (2008). Lethal Activity of Nonthermal Plasma Sterilization against Microorganisms. Infect. Control Hosp. Epidemiol..

[103] Yasuda H., Miura T., Kurita H., Takashima K., Mizuno A. (2010). Biological Evaluation of DNA Damage in Bacteriophages Inactivated by Atmospheric Pressure Cold Plasma. Plasma Process. Polym..

[104] Guo L., Xu R., Gou L., Liu Z., Zhao Y., Liu D., Zhang L., Chen H., Kong M.G. (2018). Mechanism of Virus Inactivation by Cold Atmospheric-Pressure Plasma and Plasma-Activated Water. Appl. Environ. Microbiol..

[105] Guha G. (1959). Temperature Tolerance and Kinetics of Thermal Inactivation in *E. coli* Communior Phage of Various Concentrations. Proc. Soc. Exp. Biol. Med..

[106] Lee S.J., Si J., Yun H.S., Ko G.P. (2015). Effect of Temperature and Relative Humidity on the Survival of Foodborne Viruses during Food Storage. Appl. Environ. Microbiol..

[107] Kim S.J., Si J., Lee J.E., Ko G. (2012). Temperature and Humidity Influences on Inactivation Kinetics of Enteric Viruses on Surfaces. Environ. Sci. Technol..

[108] Chen H., Joerger R.D., Kingsley D.H., Hoover D.G. (2004). Pressure Inactivation Kinetics of Phage Lambda CI 857. J. Food Prot..

[109] Lea D.E., Salaman M.H., Lea D.E., Salaman M.H. (1946). Experiments on the Inactivation of Bacteriophage by Radiations, and Their Bearing on the Nature of Bacteriophage. RSPSB.

[110] Sommer R., Pribil W., Appelt S., Gehringer P., Eschweiler H., Leth H., Cabaj A., Haider T. (2001). Inactivation of Bacteriophages in Water by Means of Non-Ionizing (UV-253.7 Nm) and Ionizing (Gamma) Radiation: A Comparative Approach. Water Res..

[111] Thompson J.E., III E.R.B. (2000). Gamma Irradiation for Inactivation of *C. parvum*, *E. coli*, and Coliphage MS-2. J. Environ. Eng..

[112] Karaböce B., Saban E., Aydın Böyük A., Okan Durmuş H., Hamid R., Baş A. (2022). Inactivation of Viruses on Surfaces by Infrared Techniques. Int. J. Therm. Sci..

[113] Bryant S., Rahmanian R., Tam H., Zabetian S. (2007). Effects Of Microwave Irradiation And Heat On T4 Bacteriophage Inactivation. J. Exp. Microbiol. Immunol. (JEMI).

[114] Nikolić B., Milojević N., Stanisavljev D., Knežević-Vukčević J. (2014). Different Effects of Microwaves and Conventional Heating on Bacteriophage and Proliferation in *E. coli*. Arch. Biol. Sci..

[115] Park D.-K., Bitton G., Melker R. (2006). Microbial Inactivation by Microwave Radiation in the Home Environment. J. Environ. Health.

[116] Yüzbasi N.S., Krawczyk P.A., Domagała K.W., Englert A., Burkhardt M., Stuer M., Graule T. (2022). Removal of MS2 and Fr Bacteriophages Using MgAl_2_O_4_-Modified, Al_2_O_3_-Stabilized Porous Ceramic Granules for Drinking Water Treatment. Membranes.

[117] Jackson K.N., Kahler D.M., Kucharska I., Rekosh D., Hammarskjold M.-L., Smith J.A. (2019). Inactivation of MS2 Bacteriophage and Adenovirus with Silver and Copper in Solution and Embedded in Ceramic Water Filters. J. Environ. Eng..

[118] Park K.T., Hwang J. (2014). Filtration and Inactivation of Aerosolized Bacteriophage MS2 by a CNT Air Filter Fabricated Using Electro-Aerodynamic Deposition. Carbon N. Y..

[119] Fina L.R., Hassouna N., Horacek G.L., Lambert J.P., Lambert J.L. (1982). Viricidal Capability of Resin-Triiodide Demand-Type Disinfectant. Appl. Environ. Microbiol..

[120] Marchin G.L., Silverstein J., Brion G.M. (1997). Effect of Microgravity on *Escherichia coli* and MS-2 Bacteriophage Disinfection by Iodinated Resins. Acta Astronaut..

[121] Lee J.H., Wu C.Y., Lee C.N., Anwar D., Wysocki K.M., Lundgren D.A., Farrah S., Wander J., Heimbuch B.K. (2009). Assessment of Iodine-Treated Filter Media for Removal and Inactivation of MS2 Bacteriophage Aerosols. J. Appl. Microbiol..

[122] Shang M., Kong Y., Yang Z., Cheng R., Zheng X., Liu Y., Chen T. (2023). Removal of Virus Aerosols by the Combination of Filtration and UV-C Irradiation. Front. Environ. Sci. Eng..

[123] Mamane H., Shemer H., Linden K.G. (2007). Inactivation of *E. coli*, *B. subtilis* Spores, and MS2, T4, and T7 Phage Using UV/H2O2 Advanced Oxidation. J. Hazard. Mater..

[124] Templeton M.R., Andrews R.C., Hofmann R. (2007). Removal of Particle-Associated Bacteriophages by Dual-Media Filtration at Different Filter Cycle Stages and Impacts on Subsequent UV Disinfection. Water Res..

[125] Jolis D., Hirano R., Pitt P. (1999). Tertiary Treatment Using Microfiltration and UV Disinfection for Water Reclamation. Water Environ. Res..

[126] Clark E.M., Wright H., Lennon K.A., Craik V.A., Clark J.R., March J.B. (2012). Inactivation of Recombinant Bacteriophage Lambda by Use of Chemical Agents and UV Radiation. Appl. Environ. Microbiol..

[127] Mamane-Gravetz H., Linden K.G., Cabaj A., Sommer R. (2005). Spectral Sensitivity of Bacillus Subtilis Spores and MS2 Coliphage for Validation Testing of Ultraviolet Reactors for Water Disinfection. Environ. Sci. Technol..

[128] Schmidt S., Kauling J. (2007). Process and Laboratory Scale UV Inactivation of Viruses and Bacteria Using an Innovative Coiled Tube Reactor. Chem. Eng. Technol..

[129] Fallon K.S., Hargy T.M., Mackey E.D., Wright H.B., Clancy J.L. (2007). Development and Characterization of Nonpathogenic Surrogates for UV Reactor Validation. J. Am. Water Works Assoc..

[130] Sommer R., Haider T., Cabaj A., Pribil W., Lhotsky M. (1998). Time Dose Reciprocity in UV Disinfection of Water. Water Sci. Technol..

[131] Malley J.P. (2004). Inactivation of Pathogens with Innovative UV Technologies.

[132] Mamane-Gravetz H., Linden K.G. (2005). Impact of Particle Aggregated Microbes and Particle Scattering on UV Disinfection. Proc. Water Environ. Fed..

[133] Bae K.S., Shin G.A. (2016). Inactivation of Various Bacteriophages by Different Ultraviolet Technologies: Development of a Reliable Virus Indicator System for Water Reuse. Environ. Eng. Res..

[134] Los M., Czyz A., Sell E., Wegrzyn A., Neubauer P., Wegrzyn G. (2004). Bacteriophage Contamination: Is There a Simple Method to Reduce Its Deleterious Effects in Laboratory Cultures and Biotechnological Factories?. J. Appl. Genet..

[135] Simonet J., Gantzer C. (2006). Inactivation of Poliovirus 1 and F-Specific RNA Phages and Degradation of Their Genomes by UV Irradiation at 254 Nanometers. Appl. Environ. Microbiol..

[136] Thurston-Enriquez J.A., Haas C.N., Jacangelo J., Riley K., Gerba C.P. (2003). Inactivation of Feline Calicivirus and Adenovirus Type 40 by UV Radiation. Appl. Environ. Microbiol..

[137] Sommer R., Weber G., Cabaj A., Wekerle J., Keck G., Schauberger G. (1989). UV-Inaktivierung von Mikroorganismen in Wasser [UV-Inactivation of Microorganisms in Water]. Zent. Hyg. Umweltmed. Int. J. Hyg. Environ. Med..

[138] Rudhart S.A., Günther F., Dapper L., Stuck B.A., Hoch S. (2022). UV-C Light-Based Surface Disinfection: Analysis of Its Virucidal Efficacy Using a Bacteriophage Model. Int. J. Environ. Res. Public Health.

[139] Vitzilaiou E., Liang Y., Castro-Mejía J.L., Franz C.M.A.P., Neve H., Vogensen F.K., Knøchel S. (2022). UV Tolerance of *Lactococcus lactis* 936-Type Phages: Impact of Wavelength, Matrix, and PH. Int. J. Food Microbiol..

[140] Beck S.E., Ryu H., Boczek L.A., Cashdollar J.L., Jeanis K.M., Rosenblum J.S., Lawal O.R., Linden K.G. (2017). Evaluating UV-C LED Disinfection Performance and Investigating Potential Dual-Wavelength Synergy. Water Res..

[141] Martino V., Ochsner K., Peters P., Zitomer D.H., Mayer B.K. (2021). Virus and Bacteria Inactivation Using Ultraviolet Light-Emitting Diodes. Environ. Eng. Sci..

[142] de Roda Husman A.M., Bijkerk P., Lodder W., van den Berg H., Pribil W., Cabaj A., Gehringer P., Sommer R., Duizer E. (2004). Calicivirus Inactivation by Nonionizing (253.7-Nanometer-Wavelength [UV]) and Ionizing (Gamma) Radiation. Appl. Environ. Microbiol..

[143] Tom E.F., Molineux I.J., Paff M.L., Bull J.J. (2018). Experimental Evolution of UV Resistance in a Phage. PeerJ.

[144] Zyara A.M., Torvinen E., Veijalainen A.M., Heinonen-Tanski H. (2016). The Effect of Chlorine and Combined Chlorine/UV Treatment on Coliphages in Drinking Water Disinfection. J. Water Health.

[145] Shang C., Cheung L.M., Liu W. (2007). MS2 Coliphage Inactivation with UV Irradiation and Free Chlorine/Monochloramine. Environ. Eng. Sci..

[146] Rattanakul S., Oguma K., Sakai H., Takizawa S. (2014). Inactivation of Viruses by Combination Processes of UV and Chlorine. J. Water Environ. Technol..

[147] Oh B.S., Jang H.Y., Jung Y.J., Kang J.W. (2007). Microfiltration of MS2 Bacteriophage: Effect of Ozone on Membrane Fouling. J. Memb. Sci..

[148] Gyürék L.L., Finch G.R. (1998). Modeling Water Treatment Chemical Disinfection Kinetics. J. Environ. Eng..

[149] Tanino T., Yoshida T., Sakai K., Bing S., Ohshima T. (2015). Inactivation of *Escherichia coli* Phages by PEF Treatment and Analysis of Inactivation Mechanism. J. Electrost..

[150] Fadeeva N.P., Rautenshtein Y.I., El&apos, Piner I.E. (1959). Effect of Ultrasonic Waves on Some Actinophages and Bacteriophages. Mikrobiol. Transl..

[151] Versoza M., Jung W., Barabad M.L., Lee Y., Choi K., Park D. (2018). Inactivation of Filter Bound Aerosolized MS2 Bacteriophages Using a Non-Conductive Ultrasound Transducer. J. Virol. Methods.

[152] Kelly-Wintenberg K., Hodge A., Montie T.C., Deleanu L., Sherman D., Roth J.R., Tsai P., Wadsworth L. (1999). Use of a One Atmosphere Uniform Glow Discharge Plasma to Kill a Broad Spectrum of Microorganisms. J. Vac. Sci. Technol. A Vac. Surf. Film..

[153] Kelly-Wintenberg K., Sherman D.M., Tsai P.P.Y., Gadri R.B., Karakaya F., Chen Z., Reece Roth J., Montie T.C. (2000). Air Filter Sterilization Using a One Atmosphere Uniform Glow Discharge Plasma (the Volfilter). IEEE Trans. Plasma Sci..

[154] Tanaka Y., Yasuda H., Kurita H., Takashima K., Mizuno A. Analysis of the Inactivation Mechanism of Bacteriophage ΦX174 by Atmospheric Pressure Discharge Plasma. Proceedings of the Conference Record—IAS Annual Meeting (IEEE Industry Applications Society).

[155] Nagar V., Kar R., Pansare-Godambe L., Chand N., Bute A., Bhale D., Rao A.V.S.S.N., Shashidhar R., Maiti N. (2022). Evaluation of Virucidal Efficacy of Cold Plasma on Bacteriophage Inside a Three-Layered Sterilization Chamber. Plasma Chem. Plasma Process..

[156] Wu Y., Liang Y., Wei K., Li W., Yao M., Zhang J., Grinshpun S.A. (2015). MS2 Virus Inactivation by Atmospheric-Pressure Cold Plasma Using Different Gas Carriers and Power Levels. Appl. Environ. Microbiol..

[157] Xia T., Kleinheksel A., Lee E.M., Qiao Z., Wigginton K.R., Clack H.L. (2019). Inactivation of Airborne Viruses Using a Packed Bed Non-Thermal Plasma Reactor. J. Phys. D Appl. Phys..

[158] Yasuda H., Hashimoto M., Rahman M.M., Takashima K., Mizuno A. (2008). States of Biological Components in Bacteria and Bacteriophages during Inactivation by Atmospheric Dielectric Barrier Discharges. Plasma Process. Polym..

[159] Berchtikou A., Sokullu E., Nahar S., Tijssen P., Gauthier M.A., Ozaki T. (2020). Comparative Study on the Inactivation of MS2 and M13 Bacteriophages Using Energetic Femtosecond Lasers. J. Biophotonics.

[160] Dykeman E.C., Sankey O.F. (2009). Vibrational Energy Funneling in Viruses-Simulations of Impulsive Stimulated Raman Scattering in M13 Bacteriophage. J. Phys. Condens. Matter..

[161] Dubuis M.E., Dumont-Leblond N., Laliberté C., Veillette M., Turgeon N., Jean J., Duchaine C. (2020). Ozone Efficacy for the Control of Airborne Viruses: Bacteriophage and Norovirus Models. PLoS ONE.

[162] Cheng X., Imai T., Teeka J., Yamaguchi J., Hirose M., Higuchi T., Sekine M. (2011). Inactivation of *Escherichia coli* and Bacteriophage T4 by High Levels of Dissolved CO_2_. Appl. Microbiol. Biotechnol..

[163] Cheng X., Imai T., Teeka J., Hirose M., Higuchi T., Sekine M. (2013). Inactivation of Bacteriophages by High Levels of Dissolved CO_2_. Environ. Technol..

[164] Swathy J.R., Udhaya Sankar M., Chaudhary A., Aigal S., Anshup, Pradeep T. (2014). Antimicrobial Silver: An Unprecedented Anion Effect. Sci. Rep..

[165] Minoshima M., Lu Y., Kimura T., Nakano R., Ishiguro H., Kubota Y., Hashimoto K., Sunada K. (2016). Comparison of the Antiviral Effect of Solid-State Copper and Silver Compounds. J. Hazard. Mater..

[166] Soliman M.Y.M., Medema G., Bonilla B.E., Brouns S.J.J., van Halem D. (2020). Inactivation of RNA and DNA Viruses in Water by Copper and Silver Ions and Their Synergistic Effect. Water Res. X.

[167] Li J., Dennehy J.J. (2011). Differential Bacteriophage Mortality on Exposure to Copper. Appl. Environ. Microbiol..

[168] Pecson B.M., Decrey L., Kohn T. (2012). Photoinactivation of Virus on Iron-Oxide Coated Sand: Enhancing Inactivation in Sunlit Waters. Water Res..

[169] Fidalgo De Cortalezzi M.M., Gallardo M.V., Yrazu F., Gentile G.J., Opezzo O., Pizarro R., Poma H.R., Rajal V.B. (2014). Virus Removal by Iron Oxide Ceramic Membranes. J. Environ. Chem. Eng..

[170] Heffron J., McDermid B., Mayer B.K. (2019). Bacteriophage Inactivation as a Function of Ferrous Iron Oxidation. Environ. Sci..

[171] Giannakis S., Liu S., Carratalà A., Rtimi S., Talebi Amiri M., Bensimon M., Pulgarin C. (2017). Iron Oxide-Mediated Semiconductor Photocatalysis vs. Heterogeneous Photo-Fenton Treatment of Viruses in Wastewater. Impact of the Oxide Particle Size. J. Hazard. Mater..

[172] Kim J.Y., Lee C., Love D.C., Sedlak D.L., Yoon J., Nelson K.L. (2011). Inactivation of MS2 Coliphage by Ferrous Ion and Zero-Valent Iron Nanoparticles. Environ. Sci. Technol..

[173] Salata O.V. (2004). Applications of Nanoparticles in Biology and Medicine. J. Nanobiotechnol..

[174] Khan I., Saeed K., Khan I. (2019). Nanoparticles: Properties, Applications and Toxicities. Arab. J. Chem..

[175] Tian H., He B., Yin Y., Liu L., Shi J., Hu L., Jiang G. (2022). Chemical Nature of Metals and Metal-Based Materials in Inactivation of Viruses. Nanomaterials.

[176] Choudhury H., Pandey M., Lim Y.Q., Low C.Y., Lee C.T., Marilyn T.C.L., Loh H.S., Lim Y.P., Lee C.F., Bhattamishra S.K. (2020). Silver Nanoparticles: Advanced and Promising Technology in Diabetic Wound Therapy. Mater. Sci. Eng. C.

[177] Bekele A.Z., Gokulan K., Williams K.M., Khare S. (2016). Dose and Size-Dependent Antiviral Effects of Silver Nanoparticles on Feline Calicivirus, a Human Norovirus Surrogate. Foodborne Pathog. Dis..

[178] Gilcrease E., Williams R., Goel R. (2020). Evaluating the Effect of Silver Nanoparticles on Bacteriophage Lytic Infection Cycle-a Mechanistic Understanding. Water Res..

[179] Gokulan K., Bekele A.Z., Drake K.L., Khare S. (2018). Responses of Intestinal Virome to Silver Nanoparticles: Safety Assessment by Classical Virology, Whole-Genome Sequencing and Bioinformatics Approaches. Int. J. Nanomed..

[180] Ko Y.S., Joe Y.H., Seo M., Lim K., Hwang J., Woo K. (2014). Prompt and Synergistic Antibacterial Activity of Silver Nanoparticle-Decorated Silica Hybrid Particles on Air Filtration. J. Mater. Chem. B.

[181] Yang X.X., Li C.M., Huang C.Z. (2016). Curcumin Modified Silver Nanoparticles for Highly Efficient Inhibition of Respiratory Syncytial Virus Infection. Nanoscale.

[182] Gahlawat G., Shikha S., Chaddha B.S., Chaudhuri S.R., Mayilraj S., Choudhury A.R. (2016). Microbial Glycolipoprotein-Capped Silver Nanoparticles as Emerging Antibacterial Agents against Cholera. Microb. Cell Fact..

[183] Park S.J., Ko Y.S., Jung H., Lee C., Woo K., Ko G.P. (2018). Disinfection of Waterborne Viruses Using Silver Nanoparticle-Decorated Silica Hybrid Composites in Water Environments. Sci. Total Environ..

[184] Shimabuku Q.L., Ueda-Nakamura T., Bergamasco R., Fagundes-Klen M.R. (2018). Chick-Watson Kinetics of Virus Inactivation with Granular Activated Carbon Modified with Silver Nanoparticles and/or Copper Oxide. Process Saf. Environ. Prot..

[185] Shimabuku Q.L., Arakawa F.S., Fernandes Silva M., Ferri Coldebella P., Ueda-Nakamura T., Fagundes-Klen M.R., Bergamasco R. (2017). Water Treatment with Exceptional Virus Inactivation Using Activated Carbon Modified with Silver (Ag) and Copper Oxide (CuO) Nanoparticles. Environ. Technol..

[186] Hijnen W.A.M., Suylen G.M.H., Bahlman J.A., Brouwer-Hanzens A., Medema G.J. (2010). GAC Adsorption Filters as Barriers for Viruses, Bacteria and Protozoan (Oo)Cysts in Water Treatment. Water Res..

[187] Richter Ł., Paszkowska K., Cendrowska U., Olgiati F., Silva P.J., Gasbarri M., Guven Z.P., Paczesny J., Stellacci F. (2021). Broad-Spectrum Nanoparticles against Bacteriophage Infections. Nanoscale.

[188] Cheng R., Li G., Cheng C., Liu P., Shi L., Ma Z., Zheng X. (2014). Removal of Bacteriophage F2 in Water by Nanoscale Zero-Valent Iron and Parameters Optimization Using Response Surface Methodology. Chem. Eng. J..

[189] Cheng R., Li G., Shi L., Xue X., Kang M., Zheng X. (2016). The Mechanism for Bacteriophage F2 Removal by Nanoscale Zero-Valent Iron. Water Res..

[190] Cheng R., Kang M., Zhuang S., Wang S., Zheng X., Pan X., Shi L., Wang J. (2018). Removal of Bacteriophage F2 in Water by Fe/Ni Nanoparticles: Optimization of Fe/Ni Ratio and Influencing Factors. Sci. Total Environ..

[191] Cheng R., Zhang Y., Zhang T., Hou F., Cao X., Shi L., Jiang P., Zheng X., Wang J. (2022). The Inactivation of Bacteriophages MS2 and PhiX174 by Nanoscale Zero-Valent Iron: Resistance Difference and Mechanisms. Front. Environ. Sci. Eng..

[192] Raza S., Folga M., Łoś M., Foltynowicz Z., Paczesny J. (2022). The Effect of Zero-Valent Iron Nanoparticles (NZVI) on Bacteriophages. Viruses.

[193] Yap C.Y., Chua C.K., Dong Z.L., Liu Z.H., Zhang D.Q., Loh L.E., Sing S.L. (2015). Review of Selective Laser Melting: Materials and Applications. Appl. Phys. Rev..

[194] Guillon O., Gonzalez-Julian J., Dargatz B., Kessel T., Schierning G., Räthel J., Herrmann M. (2014). Field-Assisted Sintering Technology/Spark Plasma Sintering: Mechanisms, Materials, and Technology Developments. Adv. Eng. Mater..

[195] Molan K., Rahmani R., Krklec D., Brojan M., Stopar D. (2022). Phi 6 Bacteriophage Inactivation by Metal Salts, Metal Powders, and Metal Surfaces. Viruses.

[196] Rahmani R., Molan K., Brojan M., Prashanth K.G., Stopar D. (2022). High Virucidal Potential of Novel Ceramic–Metal Composites Fabricated via Hybrid Selective Laser Melting and Spark Plasma Sintering Routes. Int. J. Adv. Manuf. Technol..

[197] Malato S., Fernández-Ibáñez P., Maldonado M.I., Blanco J., Gernjak W. (2009). Decontamination and Disinfection of Water by Solar Photocatalysis: Recent Overview and Trends. Catal. Today.

[198] Kumar S.G., Devi L.G. (2011). Review on Modified TiO_2_ Photocatalysis under UV/Visible Light: Selected Results and Related Mechanisms on Interfacial Charge Carrier Transfer Dynamics. J. Phys. Chem. A.

[199] Pelaez M., Nolan N.T., Pillai S.C., Seery M.K., Falaras P., Kontos A.G., Dunlop P.S.M., Hamilton J.W.J., Byrne J.A., O’Shea K. (2012). A Review on the Visible Light Active Titanium Dioxide Photocatalysts for Environmental Applications. Appl. Catal. B.

[200] Wang W., Huang G., Yu J.C., Wong P.K. (2015). Advances in Photocatalytic Disinfection of Bacteria: Development of Photocatalysts and Mechanisms. J. Environ. Sci..

[201] Ditta I.B., Steele A., Liptrot C., Tobin J., Tyler H., Yates H.M., Sheel D.W., Foster H.A. (2008). Photocatalytic Antimicrobial Activity of Thin Surface Films of TiO(2), CuO and TiO(2)/CuO Dual Layers on *Escherichia coli* and Bacteriophage T4. Appl. Microbiol. Biotechnol..

[202] Zheng X., Shen Z.P., Cheng C., Shi L., Cheng R., Yuan D.H. (2018). Photocatalytic Disinfection Performance in Virus and Virus/Bacteria System by Cu-TiO_2_ Nanofibers under Visible Light. Environ. Pollut..

[203] Syngouna V.I., Chrysikopoulos C.V. (2017). Inactivation of MS2 Bacteriophage by Titanium Dioxide Nanoparticles in the Presence of Quartz Sand with and without Ambient Light. J. Colloid Interface Sci..

[204] You Y., Han J., Chiu P.C., Jin Y. (2005). Removal and Inactivation of Waterborne Viruses Using Zerovalent Iron. Environ. Sci. Technol..

[205] Jin S.E., Jin H.E. (2021). Multiscale Metal Oxide Particles to Enhance Photocatalytic Antimicrobial Activity against *Escherichia coli* and M13 Bacteriophage under Dual Ultraviolet Irradiation. Pharmaceutics.

[206] Sunada K., Minoshima M., Hashimoto K. (2012). Highly Efficient Antiviral and Antibacterial Activities of Solid-State Cuprous Compounds. J. Hazard. Mater..

[207] Mazurkow J.M., Yüzbasi N.S., Domagala K.W., Pfeiffer S., Kata D., Graule T. (2020). Nano-Sized Copper (Oxide) on Alumina Granules for Water Filtration: Effect of Copper Oxidation State on Virus Removal Performance. Environ. Sci. Technol..

[208] Ishiguro H., Nakano R., Yao Y., Kajioka J., Fujishima A., Sunada K., Minoshima M., Hashimoto K., Kubota Y. (2011). Photocatalytic Inactivation of Bacteriophages by TiO_2_-Coated Glass Plates under Low-Intensity, Long-Wavelength UV Irradiation. Photochem. Photobiol. Sci..

[209] Doolittle M.M., Cooney J.J. (1992). Inactivation of Bacteriophage T4 by Organic and Inorganic Tin Compounds. J. Ind. Microbiol..

[210] You J., Zhang Y., Hu Z. (2011). Bacteria and Bacteriophage Inactivation by Silver and Zinc Oxide Nanoparticles. Colloids Surf. B Biointerfaces.

[211] Guglielmotti D.M., Mercanti D.J., Reinheimer J.A., del Quiberoni A.L., Gänzle M., de Souza Sant A. (2012). Review: Efficiency of Physical and Chemical Treatments on the Inactivation of Dairy Bacteriophages. Front. Microbiol..

[212] Campagna C., Villion M., Labrie S.J., Duchaine C., Moineau S. (2014). Inactivation of Dairy Bacteriophages by Commercial Sanitizers and Disinfectants. Int. J. Food Microbiol..

[213] Maillard J.Y. (1996). Damage to Pseudomonas Aeruginosa PAO1 Bacteriophage F116 DNA by Biocides. J. Appl. Bacteriol..

[214] Halfhide D.E., Gannon B.W., Hayes C.M., Roe J.M. (2008). Wide Variation in Effectiveness of Laboratory Disinfectants against Bacteriophages. Lett. Appl. Microbiol..

[215] Ly-Chatain M.H., Moussaoui S., Vera A., Rigobello V., Demarigny Y., del Luján Quiberoni A. (2013). Antiviral Effect of Cationic Compounds on Bacteriophages. Front. Microbiol..

[216] Sands J.A. (1986). Virucidal Activity of Cetyltrimethylammonium Bromide below the Critical Micelle Concentration. FEMS Microbiol. Lett..

[217] Maillard J.Y., Beggs T.S., Day M.J., Hudson R.A., Russell A.D. (1994). Effect of Biocides on MS2 and K Coliphages. Appl. Environ. Microbiol..

[218] Jetté L.P., Ringuette L., Ishak M., Miller M., Saint-Antoine P. (1995). Evaluation of Three Glutaraldehyde-Based Disinfectants Used in Endoscopy. J. Hosp. Infect..

[219] Ben-Shabat S., Yarmolinsky L., Porat D., Dahan A. (2020). Antiviral Effect of Phytochemicals from Medicinal Plants: Applications and Drug Delivery Strategies. Drug Deliv. Transl. Res..

[220] Badshah S.L., Faisal S., Muhammad A., Poulson B.G., Emwas A.H., Jaremko M. (2021). Antiviral Activities of Flavonoids. Biomed. Pharmacother..

[221] Su X., Sangster M.Y., D’Souza D.H. (2011). Time-Dependent Effects of Pomegranate Juice and Pomegranate Polyphenols on Foodborne Viral Reduction. Foodborne Pathog. Dis..

[222] Maillard J.-Y., Beggs T.S., Day M.J., Hudson R.A., Russell A.D. (1995). Effects of Biocides on the Transduction of Pseudomonas Aeruginosa PAO by F116 Bacteriophage. Lett. Appl. Microbiol..

[223] Morita J. (1988). Inactivation of Bacteriophages by Polyphenols in the Presence of Cupric Ion. Agric. Biol. Chem..

[224] Philippe C., Chaïb A., Jaomanjaka F., Cluzet S., Lagarde A., Ballestra P., Decendit A., Petrel M., Claisse O., Goulet A. (2020). Wine Phenolic Compounds Differently Affect the Host-Killing Activity of Two Lytic Bacteriophages Infecting the Lactic Acid Bacterium Oenococcus Oeni. Viruses.

[225] Silva-Beltrán N.P., Ruiz-Cruz S., Chaidez C., Ornelas-Paz J.D.J., López-Mata M.A., Márquez-Riós E., Estrada M.I. (2014). Chemical Constitution and Effect of Extracts of Tomato Plants Byproducts on the Enteric Viral Surrogates. Int. J. Environ. Health Res..

[226] Lee A., Eschenbruch R., Waller J. (2011). Effect of Phenolic Compounds, Ethyl Alcohol, and Sodium Metabisulphite on the Lytic Activity of Phage PL-1 on a Lactobacillus Casei S Strain. Can. J. Microbiol..

[227] Shinozuka K., Kikuchi Y., Nishino C., Mori A., Tawata S. (1988). Inhibitory Effect of Flavonoids on DNA-Dependent DNA and RNA Polymerases. Experientia.

[228] Yang J., Tang C.B., Xiao J., Du W.F., Li R. (2018). Influences of Epigallocatechin Gallate and Citric Acid on *Escherichia coli* O157:H7 Toxin Gene Expression and Virulence-Associated Stress Response. Lett. Appl. Microbiol..

[229] Catel-Ferreira M., Tnani H., Hellio C., Cosette P., Lebrun L. (2015). Antiviral Effects of Polyphenols: Development of Bio-Based Cleaning Wipes and Filters. J. Virol. Methods.

[230] Su X., D’Souza D.H. (2012). Inactivation of Human Norovirus Surrogates by Benzalkonium Chloride, Potassium Peroxymonosulfate, Tannic Acid, and Gallic Acid. Foodborne Pathog. Dis..

[231] Bhat R., Hadi S.M. (1994). DNA Breakage by Tannic Acid and Cu(II): Generation of Active Oxygen Species and Biological Activity of the Reaction. Mutat. Res. Environ. Mutagen. Relat. Subj..

[232] Karlsson I., Samuelsson K., Ponting D.J., Törnqvist M., Ilag L.L., Nilsson U. (2016). Peptide Reactivity of Isothiocyanates--Implications for Skin Allergy. Sci. Rep..

[233] Lin C.M., Preston J.F., Wei C.I. (2000). Antibacterial Mechanism of Allyl Isothiocyanate. J. Food Prot..

[234] Romeo L., Iori R., Rollin P., Bramanti P., Mazzon E. (2018). Molecules Isothiocyanates: An Overview of Their Antimicrobial Activity against Human Infections. Molecules.

[235] Drobnica L., Zemanová M., Nemec P., Antos K., Kristián P., Stullerová A., Knoppová V., Nemec P. (1967). Antifungal Activity of Isothiocyanates and Related Compounds. I. Naturally Occurring Isothiocyanates and Their Analogues. Appl. Microbiol..

[236] Wiczk A., Hofman D., Konopa G., Herman-Antosiewicz A. (2012). Sulforaphane, a Cruciferous Vegetable-Derived Isothiocyanate, Inhibits Protein Synthesis in Human Prostate Cancer Cells. Biochim. Biophys. Acta.

[237] Singh S.V., Singh K. (2012). Cancer Chemoprevention with Dietary Isothiocyanates Mature for Clinical Translational Research. Carcinogenesis.

[238] Nowicki D., Rodzik O., Herman-Antosiewicz A., Szalewska-Pałasz A. (2016). Isothiocyanates as Effective Agents against Enterohemorrhagic *Escherichia coli*: Insight to the Mode of Action. Sci. Rep..

[239] Nowicki D., Macia̧g-Dorszyńska M., Kobiela W., Herman-Antosiewicz A., Wȩgrzyn A., Szalewska-Pałasz A., Wȩgrzyn G. (2014). Phenethyl Isothiocyanate Inhibits Shiga Toxin Production in Enterohemorrhagic *Escherichia coli* by Stringent Response Induction. Antimicrob. Agents Chemother..

[240] Potrykus K., Cashel M. (2008). (P)PpGpp: Still Magical?. Annu. Rev. Microbiol..

[241] Łoś M., Golec P., Łoś J.M., Weglewska-Jurkiewicz A., Czyz A., Wegrzyn A., Wegrzyn G., Neubauer P. (2007). Effective Inhibition of Lytic Development of Bacteriophages λ, P1 and T4 by Starvation of Their Host, *Escherichia coli*. BMC Biotechnol..

[242] Potrykus K., Wegrzyn G., Hernandez V.J. (2002). Multiple Mechanisms of Transcription Inhibition by PpGpp at the Lambdap(R) Promoter. J. Biol. Chem..

[243] Nowicki D., Kobiela W., Wegrzyn A., Wegrzyn G., Szalewska-Palasz A. (2013). PpGpp-Dependent Negative Control of DNA Replication of Shiga Toxin-Converting Bacteriophages in *Escherichia coli*. J. Bacteriol..

[244] Alexandre E.M.C., Silva S., Santos S.A.O., Silvestre A.J.D., Duarte M.F., Saraiva J.A., Pintado M. (2019). Antimicrobial Activity of Pomegranate Peel Extracts Performed by High Pressure and Enzymatic Assisted Extraction. Food Res. Int..

[245] Tayyarcan E.K., Acar Soykut E., Menteş Yılmaz O., Boyaci I.H., Khaaladi M., Fattouch S. (2019). Investigation of Different Interactions between Staphylococcus Aureus Phages and Pomegranate Peel, Grape Seed, and Black Cumin Extracts. J. Food Saf..

[246] Su X., Sangster M.Y., D’Souza D.H. (2010). In Vitro Effects of Pomegranate Juice and Pomegranate Polyphenols on Foodborne Viral Surrogates. Foodborne Pathog. Dis..

[247] Su X., D’Souza D.H. (2011). Grape Seed Extract for Control of Human Enteric Viruses. Appl. Environ. Microbiol..

[248] Stewart S., Jassim J., Denyer D., Newby N., Linley L., Dhir D. (1998). The Specific and Sensitive Detection of Bacterial Pathogens within 4 h Using Bacteriophage Amplification. J. Appl. Microbiol..

[249] Kamimoto M., Nakai Y., Tsuji T., Shimamoto T., Shimamoto T. (2014). Antiviral Effects of Persimmon Extract on Human Norovirus and Its Surrogate, Bacteriophage MS2. J. Food Sci..

[250] Lipson S.M., Sethi L., Cohen P., Gordon R.E., Tan I.P., Burdowski A., Stotzky G. (2007). Antiviral Effects on Bacteriophages and Rotavirus by Cranberry Juice. Phytomedicine.

[251] Su X., Howell A.B., D’Souza D.H. (2010). Antiviral Effects of Cranberry Juice and Cranberry Proanthocyanidins on Foodborne Viral Surrogates—A Time Dependence Study in Vitro. Food Microbiol..

[252] Silva-Beltrán N.P., Chaidez-Quiroz C., López-Cuevas O., Ruiz-Cruz S., López-Mata M.A., Del-Toro-sánchez C.L., Marquez-Rios E., Ornelas-Paz J.D.J. (2017). Phenolic Compounds of Potato Peel Extracts: Their Antioxidant Activity and Protection against Human Enteric Viruses. J. Microbiol. Biotechnol..

[253] Kronheim S., Daniel-Ivad M., Duan Z., Hwang S., Wong A.I., Mantel I., Nodwell J.R., Maxwell K.L. (2018). A Chemical Defence against Phage Infection. Nature.

[254] Hardy A., Kever L., Frunzke J. (2022). Antiphage Small Molecules Produced by Bacteria—Beyond Protein-Mediated Defenses. Trends Microbiol..

[255] Nakamura S., Nii F., Shimizu M., Watanabe I. (1971). Inhibition of Phage Growth by an Antibiotic Rugulosin Isolated from Myrothecium Verucaria I. Properties of the Anti-Phage Effect1. J. Microbiol..

[256] Zuo P., Yu P., Alvarez P.J.J. (2021). Aminoglycosides Antagonize Bacteriophage Proliferation, Attenuating Phage Suppression of Bacterial Growth, Biofilm Formation, and Antibiotic Resistance. Appl. Environ. Microbiol..

[257] Brock T.D. (1962). The Inhibition of an RNA Bacteriophage by Streptomycin, Using Host Bacteria Resistant to the Antibiotic. Biochem. Biophys. Res. Commun..

[258] Brock T.D., Mosser J., Peacher B. (1963). The Inhibition by Streptomycin of Certain Streptococcus Bacteriophages, Using Host Bacteria Resistant to the Antibiotic. Microbiology.

[259] Jiang Z., Wei J., Liang Y., Peng N., Li Y. (2020). Aminoglycoside Antibiotics Inhibit Mycobacteriophage Infection. Antibiotics.

[260] Kever L., Hardy A., Luthe T., Hünnefeld M., Gätgens C., Milke L., Wiechert J., Wittmann J., Moraru C., Marienhagen J. (2022). Aminoglycoside Antibiotics Inhibit Phage Infection by Blocking an Early Step of the Infection Cycle. mBio.

[261] Watanabe M., August J.T. (1968). Replication of RNA Bacteriophage R23: II. Inhibition of Phage-Specific RNA Synthesis by Phleomycin. J. Mol. Biol..

[262] Post L., Price A.R. (1975). Inhibition of Bacteriophage PBS2 Replication in Bacillus Subtilis by Phleomycin. J. Virol..

[263] Cloos J., Gille J.J.P., Steen I., Lafleur M.V.M., Retèl J., Snow G.B., Braakhuis B.J.M. (1996). Influence of the Antioxidant N-Acetylcysteine and Its Metabolites on Damage Induced by Bleomycin in PM2 Bacteriophage DNA. Carcinogenesis.

[264] Haidle C.W., Weiss K.K., Mace M.L. (1972). Induction of Bacteriophage by Bleomycin. Biochem. Biophys. Res. Commun..

[265] Sato K., Niinomi Y., Katagiri K., Matsukage A., Minagawa T. (1969). Prevention of Phage Multiplication by Quinomycin A. Biochim. Biophys. Acta Nucleic Acids Protein Synth..

[266] Cieślik M., Harhala M., Orwat F., Dąbrowska K., Górski A., Jończyk-Matysiak E. (2022). Two Newly Isolated Enterobacter-Specific Bacteriophages: Biological Properties and Stability Studies. Viruses.

[267] Hübner N.O., Siebert J., Kramer A. (2010). Octenidine Dihydrochloride, a Modern Antiseptic for Skin, Mucous Membranes and Wounds. Skin Pharmacol. Physiol..

[268] Maillard J.-Y., Beggs T.S., Day M.J., Hudson R.A., Russell A.D. (1993). Effect of Biocides on Pseudomonas Aeruginosa Phage F116. Lett. Appl. Microbiol..

[269] Khatibi S.A., Misaghi A., Moosavy M.H., Akhondzadeh Basti A., Mohamadian S., Khanjari A. (2018). Effect of Nanoliposomes Containing Zataria Multiflora Boiss. Essential Oil on Gene Expression of Shiga Toxin 2 in *Escherichia coli* O157:H7. J. Appl. Microbiol..

[270] Kim S.S., Kang D.H. (2017). Synergistic Effect of Carvacrol and Ohmic Heating for Inactivation of *E. coli* O157:H7, S. Typhimurium, L. Monocytogenes, and MS-2 Bacteriophage in Salsa. Food Control.

[271] Sheng L., Rasco B., Zhu M.J. (2016). Cinnamon Oil Inhibits Shiga Toxin Type 2 Phage Induction and Shiga Toxin Type 2 Production in *Escherichia coli* O157:H7. Appl. Environ. Microbiol..

[272] Kim Y.G., Lee J.H., Kim S.I., Baek K.H., Lee J. (2015). Cinnamon Bark Oil and Its Components Inhibit Biofilm Formation and Toxin Production. Int. J. Food Microbiol..

[273] Brownell G.H., Crockett J.K. (1971). Inactivation of Nocardiophages ΦC and ΦEC by Extracts of Bacteriophage-Attachable Cells. J. Virol..

[274] Davis R., Zivanovic S., Michael Davidson P., D’Souza D.H. (2015). Enteric Viral Surrogate Reduction by Chitosan. Food Environ. Virol..

[275] Kochkina Z., Mikrobiologiia S.C. (2000). Effect of Chitosan Derivatives on the Development of Phage Infection in Cultured Bacillus Thuringiensis. Mikrobiologiia.

[276] Amorim J.H., del Cogliano M.E., Fernandez-Brando R.J., Bilen M.F., Jesus M.R., Luiz W.B., Palermo M.S., Ferreira R.C.C., Servat E.G., Ghiringhelli P.D. (2014). Role of Bacteriophages in STEC Infections: New Implications for the Design of Prophylactic and Treatment Approaches. F1000Research.

[277] de Siqueira R.S., Dodd C.E.R., Rees C.E.D. (2006). Evaluation of the Natural Virucidal Activity of Teas for Use in the Phage Amplification Assay. Int. J. Food Microbiol..

[278] Su X., Howell A.B., D’Souza D.H. (2010). The Effect of Cranberry Juice and Cranberry Proanthocyanidins on the Infectivity of Human Enteric Viral Surrogates. Food Microbiol..

[279] Liao N., Sun L., Wang D., Chen L., Wang J., Qi X., Zhang H., Tang M., Wu G., Chen J. (2021). Antiviral Properties of Propolis Ethanol Extract against Norovirus and Its Application in Fresh Juices. LWT.

[280] Magnavacca A., Sangiovanni E., Racagni G., Dell’Agli M. (2022). The Antiviral and Immunomodulatory Activities of Propolis: An Update and Future Perspectives for Respiratory Diseases. Med. Res. Rev..

[281] Silva-Beltrán N.P., Balderrama-Carmona A.P., Umsza-Guez M.A., Souza Machado B.A. (2020). Antiviral Effects of Brazilian Green and Red Propolis Extracts on Enterovirus Surrogates. Environ. Sci. Pollut. Res. Int..

[282] Richter H.E., Loewen P.C. (1982). Rapid Inactivation of Bacteriophage T7 by Ascorbic Acid Is Repairable. Biochim. Biophys. Acta Gene Struct. Expr..

[283] Kobayashi S., Ueda K., Morita J., Sakai H., Komano T. (1988). DNA Damage Induced by Ascorbate in the Presence of Cu^2+^. Biochim. Biophys. Acta Gene Struct. Expr..

[284] Murata A., Oyadomari R., Ohashi T., Kitagawa K. (1975). Mechanism of Inactivation of Bacteriophage DeltaA Containing Single-Stranded DNA by Ascorbic Acid. J. Nutr. Sci. Vitaminol..

[285] Price A.R., Fogt S.M. (1973). Effect of Nalidixic Acid on PBS2 Bacteriophage Infection of Bacillus Subtilis. J. Virol..

